# Determinants of drug-target interactions at the single cell level

**DOI:** 10.1371/journal.pcbi.1006601

**Published:** 2018-12-20

**Authors:** Vlad Elgart, Jia-Ren Lin, Joseph Loscalzo

**Affiliations:** 1 Laboratory for Systems Pharmacology, Harvard Medical School, Boston, Massachusetts, United States of America; 2 Department of Medicine, Brigham and Women’s Hospital, Boston, Massachusetts, United States of America; Bogazici University, TURKEY

## Abstract

The physiochemical determinants of drug-target interactions in the microenvironment of the cell are complex and generally not defined by simple diffusion and intrinsic chemical reactivity. Non-specific interactions of drugs and macromolecules in cells are rarely considered formally in assessing pharmacodynamics. Here, we demonstrate that non-specific interactions lead to very slow incorporation kinetics of DNA binding drugs. We observe a rate of drug incorporation in cell nuclei three orders of magnitude slower than *in vitro* due to anomalous drug diffusion within cells. This slow diffusion, however, has an advantageous consequence: it leads to virtually irreversible binding of the drug to specific DNA targets in cells. We show that non-specific interactions drive slow drug diffusion manifesting as slow reaction front propagation. We study the effect of non-specific interactions in different cellular compartments by permeabilization of plasma and nuclear membranes in order to pinpoint differential compartment effects on variability in intracellular drug kinetics. These results provide the basis for a comprehensive model of the determinants of intracellular diffusion of small-molecule drugs, their target-seeking trajectories, and the consequences of these processes on the apparent kinetics of drug-target interactions.

## Author summary

Small-molecule drug design assumes target binding of high affinity. Most small molecules can interact with other macromolecules in the cell ‘nonspecifically,’ i.e., with significantly lower affinity. The extent to which these nonspecific interactions influence the availability and action of the drug for its specific target depends upon the relative concentrations of drug, the specific target, and nonspecific targets. The structure of the cell is quite crowded with a highly non-uniform distribution of macromolecules that can interact with the drug of interest both specifically and nonspecifically. Thus, some compartments or micro-domains within the cell may have a comparatively high concentration of nonspecific targets, sufficient to trap the drug and retard its diffusion toward the specific target. Here, using small-molecule binding to DNA and single cell monitoring, we demonstrate that this effect results in apparently anomalous small molecule-DNA binding kinetics in cells at rates that are 1000-fold slower than in a homogeneous, dilute, aqueous environment. This slow intracellular diffusion, however, has an advantageous consequence: it leads to virtually irreversible binding of the small molecule (drug) to specific DNA targets in cells. We study and quantify the effect of nonspecific interactions between small DNA-binding molecules, including known DNA-binding drugs, in different cellular compartments in order to identify factors that account for the variability in binding kinetics among individual cells.

## Introduction

Drug efficacy *in vivo* is notoriously difficult to predict owing, in part, to the complexity of the underlying biochemical processes that govern drug–target interactions *in situ*. Semi-empiric pharmacokinetic/pharmacodynamic (PK/PD) models typically describe accumulation of the drug in tissue(s) and, hence, do not address the question of variability in efficacy for individual cells, which is determined by the drug’s access to and interaction with its target(s) within a cell. Variability in drug efficacy may, therefore, be a key factor driving resistance, selection, and toxicity.

Here, we investigate factors affecting drug–target interactions at the single cell level. Our model system is a monolayer cell culture that allows continuous monitoring of drug binding to its target in individual cells. While this model system is, of course, far from ideal, provided that the free drug concentration in a given tissue is fairly uniform, cell culture experiments can meaningfully address the question of heterogeneity of response in a cell population.

We monitor the kinetics of 2’-[4-ethoxyphenyl]-5-[4-methyl-1-piperazinyl]-2,5’-bi-1H-benzimidazole trihydrochloride trihydrate (Hoechst 33342 dye) incorporation in individual cell nuclei by measuring the dye’s fluorescence signal intensity. Hoechst dye becomes significantly more fluorescent upon binding to the minor groove of DNA and, therefore, fluorescence intensity corresponds to the amount of bound target (DNA) in the nucleus.

Fluorescence microscopy permits resolution of both the temporal and spatial dependence of dye incorporation. It is instructive to investigate the incorporation process on two different spatial scales. By integrating out spatial degrees of freedom, we can assess overall dye incorporation kinetics with measurement of fluorescence intensity over time, *I*_*tot*_(*t*), for individual cells. At a sub-nuclear scale, we can analyze the time dependence of individual pixel intensities, $I(\vec{x},t)$, that typically correspond to a spatial resolution two orders of magnitude smaller than the whole nucleus in our system. Individual pixel intensities are noisy, for which reason we developed a method based on moments of distribution to characterize drug diffusion and signal ‘homogenization’ within the nucleus.

We introduce a physical multi-compartment model of drug diffusion and binding/dissociation that can explain our experimental findings. Within this model, we also incorporate the effects of membrane permeability and partitioning (as recently addressed [1]). We further extend this reaction scheme to include diffusion [2–4] and account for non-specific interactions (high capacity, low affinity) between drug and macromolecules other than intended targets.

Non-specific interactions are often driven by chemical reactions requiring close proximity of interacting species. In a crowded intracellular environment with high local concentrations of non-specific binders, this proximity can be achieved. We, therefore, incorporated non-specific binding and dissociation processes into our reaction-diffusion model.

With this detailed model, we show computationally that owing to their spatial localization in the intracellular environment, non-specific binders act as a trap, reducing extracellular drug concentration and retarding specific drug-target kinetics. The implications of these findings for drug-target interactions and pharmacological efficacy are discussed.

## Materials and methods

### Cell culture

We used the MFC10A cell line with the NLS-Venus (nuclear) reporter for microscopy. Human epitheloid cervical carcinoma cells (HeLa cell line) were used for spectrofluorimetric measurements.

### Spectrofluorimetry

We used a spectroflurorimetric plate reader (SpectraMax Gemini) to monitor binding kinetics on a cell population-average level. To this end, HeLa cells were fixed with 4% formalin and resuspended in Dulbecco’s phosphate-buffered saline (dPBS, Sigma-D5652) at various cell densities. Next, cells were incubated with Hoechst 33342 dye (Invitrogen-H1399), and fluorescence changes over time were monitored using the microplate reader (excitation 350 *nm*, emission 461 *nm*). In order to measure free dye concentration in solution, cells were centrifuged and the collected supernatant was incubated with calf thymus DNA. Using a DNA standard (calf thymus, Sigma-D1501), we estimated the free dye concentration in the supernatant as a function of concentration and time.

### Microscopy

Fluorescent images were taken with the Operetta High Content Imaging System (Perkin Elmer). The 20x objective was used throughout the experiments unless otherwise noted. Image processing and analysis were performed using customized imageJ and Matlab scripts (S1 Text). In brief, the cherry-NLS signals were binarized and segmented in order to generate nuclear masks, which were applied to the Hoechst channel to obtain pixel intensities. Single-cell tracking for time-lapse experiments was archived with Python/Perl/Matlab scripts.

### Drug efficacy

Doxorubicin efficacy at the single cell level can be measured in terms of DNA damage biomarker(s), such as histone *γ*-H2Ax. In order to combine kinetic measurements in live cells with antibody staining for the *γ*-H2Ax marker, we performed immunofluorescence microscopy experiments as follows: Live cells were incubated with both Hoechst dye and doxorubicin at different concentrations and imaged for relatively short periods (typically three hours) that were sufficient to detect dynamic patterns in fluorescence staining. Immediately thereafter, cells were fixed with paraformaldehyde, stained with an anti-*γ*-H2Ax antibody, and again imaged (see Movie C in S1 File). This protocol allowed us to combine both the dynamic measurement of dye incorporation and the resulting phenotype (extent of DNA damage) for individual cells.

### Image analysis

Fluorescence images were analyzed using custom-designed in-house programs. Briefly, the image background was subtracted using ImageJ; and nuclear segmentation, tracking, and data analysis were performed using custom MATLAB code.

### Numerical simulations

Wolfram Mathematica was used to simulate reaction–diffusion model(s).

### Spatial distribution of bound dye in individual nuclei

Since MFC10A cells are fairly symmetric and ellipsoidal in shape, we can identify principal axes and positions of the ‘center of mass’ using the nuclear localization sequence marker (NLS-mCherry) as a reference (N.B., NLS fluorescence intensity is stable and unchanging over the time course of these experiments).

We introduced the distance *r* of any given pixel from the center of mass in the *xy* plane. The corresponding time dependent pixel intensity is $I_{r}\left(\theta ,t\right)=I\left(\vec{x},t\right)$ and depends, of course, on the orientation *θ* of the pixel, as well. If the target (DNA) distribution were symmetric in the nucleus and the shape of the nucleus were spherical, one would expect that all pixels positioned the same distance *r* away from the center of the nucleus would have identical dye incorporation kinetics. Similarly, for a symmetric nuclear ellipse, pixels in the *xy* plane satisfy the condition:

$$\begin{array}{c} \frac{x^{2}}{a^{2}}+\frac{y^{2}}{b^{2}}=const=r^{2} \end{array}$$ (1)

and would be expected to have identical intensities at any given time (here *a*, *b* are principal axes of the nucleus).

In reality, owing to a non-homogeneous target distribution and other factors affecting dye mobility and dye transport, pixel intensities are not identical and are noisy. Averaging over all pixels that satisfy the geometric condition of Eq (1) yields a much more robust time-dependent observable variable *I*_*r*_(*t*) = 〈*I*_*r*_(*θ*, *t*)〉 where averaging is performed over orientation angle *θ*.

We note that the actual measured quantities correspond to the integrated intensity in the *z*-dimension within the depth of the confocal plane. We take this fact into account while matching experimental and theoretical results (see S1 Text for more details).

Finally, we defined moments of the pixel intensity distribution as follows:

$$\begin{array}{c} M_{n}\left(t\right)=\frac{\sum_{j}I\left(j,t\right)r_{j}^{n}}{\sum_{j}I\left(j,t\right)} \end{array}$$ (2)

where *I*(*j*, *t*) and *r*_*j*_ are, respectively, time-dependent fluorescence intensity and distance from the center of mass for pixel *j*. This representation of the front is robust and can be defined for any nuclear geometry. This method is often used in image processing and usually referred to as the image moment method. The main advantage of this method in our case is its invariance with respect to translation, scale, and rotation [5, 6] due to movements of the cell and microscope stage.

## Results

### Time course of dye binding

**Fig 1:**
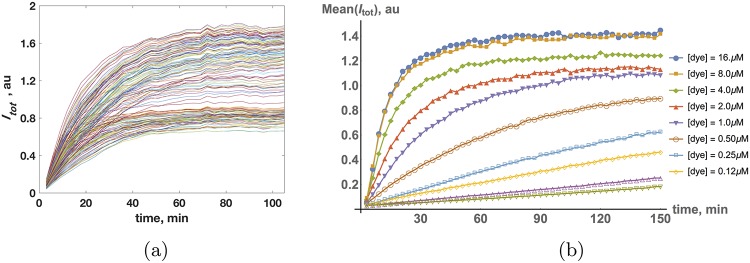
(a) Recorded time traces of overall dye incorporation, *I*_*tot*_(*t*), of individual live cells. The dye concentration is [dye] = 2 *μM*. (b) Population average of incorporation kinetics, Mean(*I*_*tot*_), for different Hoechst dye concentrations in cell culture.

Time traces of overall dye intensity (incorporation), *I*_*tot*_(*t*), for a typical experiment in live cells are depicted in Fig 1a. There are two striking features of these traces: (i) the characteristic time scale of drug incorporation kinetics, and (ii) the broad population distribution in individual cell kinetics. The dynamics of Hoechst dye incorporation for a typical cell (population average) is depicted in Fig 1b for various dye concentrations.

The time scale of 10^3^ *sec* for micromolar dye concentrations is rather unexpected based on first principles, which we next address. The simplest way to describe dye incorporation is to assume that the kinetics is driven by second order binding and first order dissociation reactions:

$$\begin{array}{c} \frac{d}{dt}\;v\left(t\right)=-{\tilde{k}}_{\text{on}}\;u\left(t\right)\;v\left(t\right)+{\tilde{k}}_{\text{off}}\;\left[c-v\left(t\right)\right] \end{array}$$ (3)

where *v* and *u* are free target and drug concentrations, respectively, and *c* is the concentration of available binding sites (capacity). The parameters ${\tilde{k}}_{\text{on}}$ and ${\tilde{k}}_{\text{off}}$ correspond to effective association and dissociation rates, respectively. These parameters depend not only on the intrinsic reaction rates, but also on the spatial disposition of the target molecules, potential competing binding targets, obstructive barriers to free diffusion, cell membrane properties, and active transport processes in the cell. It is a straightforward exercise to demonstrate that experimentally observed values of ${\tilde{k}}_{\text{on}}$ and ${\tilde{k}}_{\text{off}}$ are very different from the corresponding intrinsic values *k*_on_ and *k*_off_. Let us assume that the extracellular dye concentration is constant over time, *u*(*t*) = *u*_0_ (we will see below that this is not always the case). Under this condition, one readily derives from Eq (3) the following equation:

$$\begin{array}{c} v\left(t\right)=v_{st}+\left(c-v_{st}\right)e^{-\beta \;t} \end{array}$$ (4)

$$\begin{array}{c} \beta ={\tilde{k}}_{\text{on}}\;u_{0}+{\tilde{k}}_{\text{off}}={\tilde{k}}_{\text{off}}\;\left(1+u_{0}/K_{d}\right) \end{array}$$ (5)

$$\begin{array}{c} v_{st}=\frac{{\tilde{k}}_{\text{off}}}{\beta }\;c \end{array}$$ (6)

with the steady-state dye concentration *v*_*st*_, dissociation constant *K*_*d*_, and relaxation rate *β*. The intrinsic dissociation rate and dissociation constant for dye-DNA complexes in vitro (in cell free systems) have been measured by several groups [7, 8]:

$$\begin{array}{c} k_{\text{off}}>10^{-1}sec^{-1} \end{array}$$ (7)

$$\begin{array}{c} K_{d}<10^{-8}\;M \end{array}$$ (8)

Based on these intrinsic parameters, one would, therefore, expect a relaxation rate *β* faster than 10^−1^ *sec*^−1^ for any dye concentration *u*_0_. For a dye concentration in the micromolar range, *u*_0_ ∼ 1 *μM*, the relaxation rate is dominated by the binding reaction and would be expected to be 10 *sec*^−1^. Experimentally, however, we observed a much slower relaxation rate, of the order of 10^−3^ *sec*^−1^ (Fig 1a and 1b). We note that replacing the intrinsic association rate *k*_on_ with a conventional diffusion-driven association rate does not explain the slowness of the observed relaxation rate. First, the exponent *β* is a sum of two terms [see Eq (5)]. Second, a typical value for a diffusion-driven association rate for a small molecule the size of the dye interacting with DNA (in water) is 10^9^ *M*^−1^ *sec*^−1^, an order of magnitude faster than the intrinsic observed association rate, *k*_on_. In order to eliminate factors related to evolving cell phenotype in culture (i.e., cell fate), we also fixed cells with paraformaldehyde and measured fluorescence over an extended period of time. Of note, we observed no significant effect of fixation on the dynamics of the population average by comparing the fluorescence of live and fixed cells for time periods of less than 3 hours.

The time traces of dye incorporation are shown in Figures Aa and Ab in S1 Text for dye concentrations of 8 *μg*/*ml*. Here, we used digitonin (Fig. Aa) selectively or in combination with Triton X-100 (Fig. Ab) to permeabilize either the plasma membrane alone or all cell membranes, respectively [9]. The results (Fig. Aa) show that mild digitonin treatment at moderate dye concentrations does not affect incorporation kinetics. Digitonin at high concentration (50 ug/ml or Triton X-100 (0.1%) treatment), however, has a major impact on incorporation kinetics compared to the presence of an intact nuclear membrane (Fig. Ab). We observed acceleration in the initial phase of the incorporation rate by 2.5 − 3.5 -fold with a high concentration of digitonin or with Triton X-100 treatment of fixed cells. Nevertheless, even under these conditions, the incorporation kinetics is very slow compared to *in vitro* behavior. Since it has been reported [9] that even 5 *μg*/*ml* digitonin is sufficient to permeabilize the plasma membrane in HeLa cells, we hypothesized that the reason for accelerated kinetics in the presence of higher concentrations of detergents might not only be a consequence of dissolution of limiting membrane structures, but also dissolution of other membrane structures in the cell under these conditions.

We next assessed the effective dissociation rate of dye from cellular DNA by means of ‘cold chase’ experiments. After overnight incubation with dye, cells were centrifuged and the supernatant containing unbound dye aspirated and replaced with dPBS, after which fluorescence intensity was monitored over time. The resulting decay in fluorescence is depicted in Figure B in S1 Text. Here we compare the fluorescence intensity of cells that were chased with dye-free PBS (dPBS) (Fig. Ba) to cells that were maintained in dye-containing solution (Fig. Bb). Note that fluorescence decay was essentially unaffected by the presence of free dye in solution. The slight and near equivalent fluorescence decay in both conditions is most likely due to dye degradation at room temperature and not dissociation from DNA.

We observed that effectively irreversible tight binding of dye, resulting in fluorescence, occurs only in intact nuclei (Figure C in S1 Text). Here, lysed cells were incubated with dye, and after achieving steady-state fluorescence, chased with dye-free buffer as described above. Unlike intact cells, the fluorescence intensity of the cell lysate decreases instantaneously (on the time scale of our typical experiments) after the chase and quickly equilibrates at a new steady-state level.

### Spatial distribution of dye binding in individual nuclei

**Fig 2:**
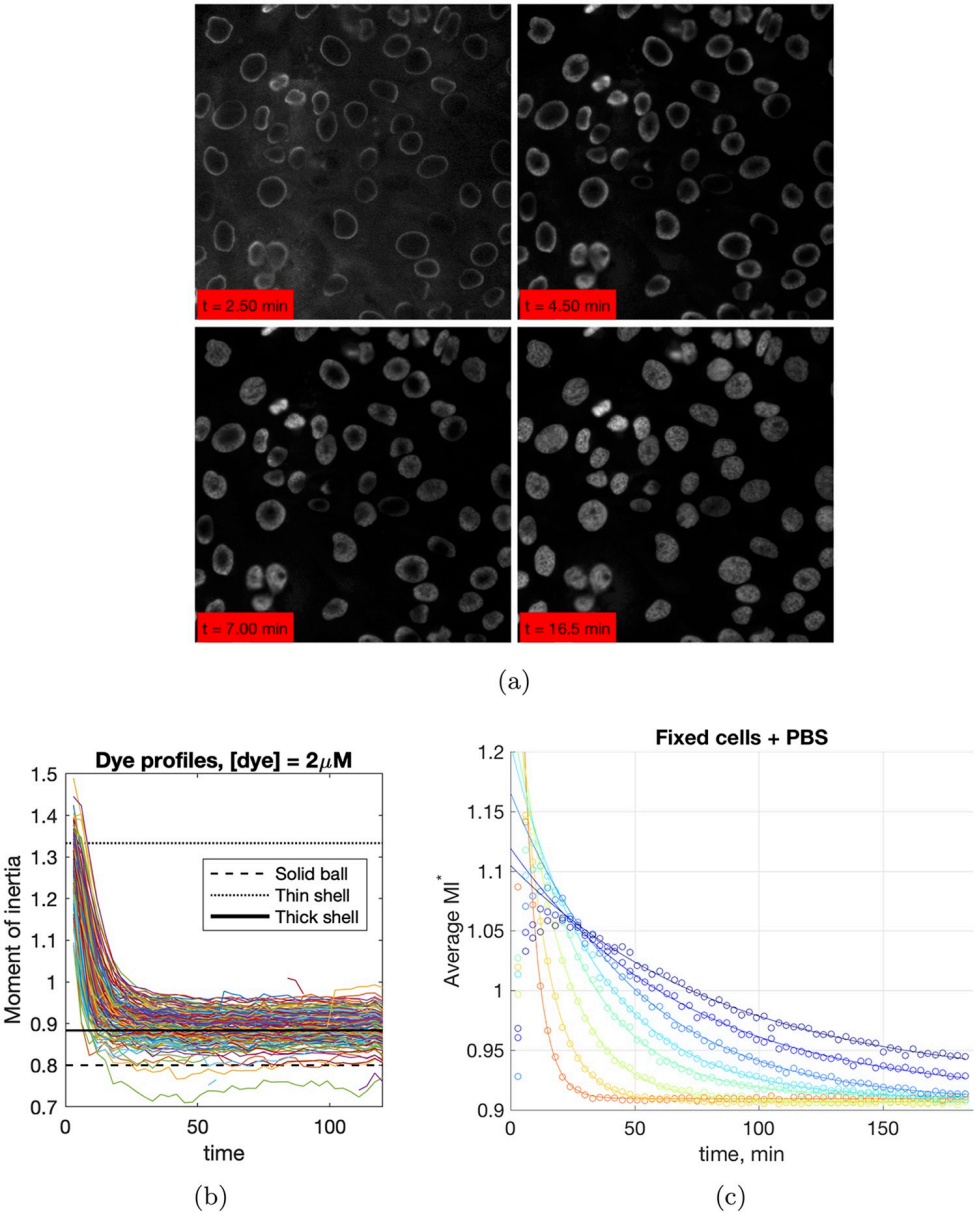
(a) Reaction front propagation during staining of MFC10A cells with Hoechst dye. (b) Time traces of moment of inertia, *M*_2_, for individual cells, [dye] = 2 *μM*, live cells. The reference lines correspond to the theoretical calculation of *M*_2_ for different geometric objects (cf. S1 Text for details). Initially, bound dye is expected to be localized in the thin outer shell of the nucleus. If the distribution of bound sites were uniform throughout a spheroidal nucleus, the expected *M*_2_ should correspond to that of a solid ball. The thick shell in the reference plot corresponds to (1/4)*R* thickness. (c) Time traces of *M*_2_ for different dye concentrations, population average, fixed cells.

In order to tease out factors contributing to the slow kinetics of dye incorporation, we studied the spatial distribution of bound dye as a function of time. Surprisingly, we observed a reaction front propagation in live cells that lasted several minutes (cf. Fig 2a, and Movies (A, B) in S1 File).

The dependence of *I*_*r*_(*t*) as a function of time is shown in Figure D in S1 Text for a typical spheroidal nucleus with principal axes of the nucleus *a* ≈ *b*. It is clear from the results of Figure D that dye incorporation dynamics is non-uniform (at least during the initial several minutes of monitoring). The observed front is a result of faster incorporation of the dye at the periphery of the nucleus compared with the center. While this is rather expected behavior, what is surprising, once again, is the kinetics of front propagation. Free dye diffusion in water is characterized by an estimated diffusion constant of 500 *μM*^2^ *sec*^−1^ [10] and, hence, the expected homogenization time in a nucleus of radius 20 *μM* is 1 *sec*, two to three orders of magnitude faster than what we observed experimentally.

Note that the results in Figure D suggest that after an initial period of homogenization (i.e., completed front propagation), the kinetics becomes uniform across the entire nucleus. To make this observation more apparent, we compared fluorescence intensities of the whole nucleus and sub-regions of the nucleus at different time points in Figure E in S1 Text (see S1 Text for the computational details). Here, a sub-region corresponds to 10% of all pixels situated around the geometric center of each individual nucleus (sub-regions were defined by “shrinking” the nucleus’s shape in each dimension proportionately and, hence, preserving nuclear geometry).

Comparison of sub-regional to total fluorescence intensity, indeed, demonstrates slow reaction front propagation dynamics that varies among cells. However, quantification of the dynamics based on this representation relies heavily on a uniform distribution of target density and symmetry of the nuclei.

A more direct and rigorous way to quantify and characterize front propagation is to calculate moments of the pixel intensity distribution *M*_*n*_, a parameter that is not dependent on symmetries in geometry and target distribution. Typical time traces of the second moment *M*_2_ are depicted in Fig 2b for individual nuclei ([dye] = 2 *μM*) and for the population average (Fig 2c, different dye concentrations). Front propagation initially drives a large second moment (only a thin shell of the nucleus incorporates dye) towards a steady-state that depends on the DNA distribution. While the typical time scale of homogenization is significantly faster than the relaxation time for overall dye intensity, it is still much slower than the 1 *sec* time scale discussed above. Note that time traces of *M*_2_ depicted in Fig 2b display variability in both relaxation kinetics and the steady–state achieved, similar to total dye incorporation *I*_*tot*_. Furthermore, we observed excellent correlation between the relaxation rates of *M*_2_ and *I*_*tot*_ time traces (cf. Fig. F in S1 Text).

### Reaction-diffusion model

The observed pattern of front propagation and incorporation suggests that the slow kinetics is driven by slow mixing of the dye in the nucleus. To confirm this hypothesis, we introduce a reaction–diffusion model that takes into account the interaction between dye and DNA, and diffusion of free dye. Assuming that DNA binding sites are largely stationary compared to dye molecules, we derive:

$$\begin{array}{c} R\left(u,v\right)=k_{\text{on}}\;u\;v-k_{\text{off}}\;\left(c-v\right) \end{array}$$ (9)

$$\begin{array}{c} \partial_{t}u\left(x,t\right)=D\;\nabla_{x}^{2}u-R\left(u,v\right) \end{array}$$ (10)

$$\begin{array}{c} \partial_{t}v\left(x,t\right)=-R\left(u,v\right) \end{array}$$ (11)

Here, R is a local reaction rate and *D* corresponds to the diffusion coefficient of free dye in the nucleus. Note that the model implicit in Eqs (9)–(11) corresponds to a mean field description and, therefore, is not suitable for the study of variability in incorporation dynamics across the nucleus. Eqs (9)–(11) also need to be supplemented by the appropriate boundary condition:

$$\begin{array}{c} D\nabla_{x}u\left(x,t\right)=h_{m}[u_{ext}-u\left(x,t\right)],\;x\in \Omega \end{array}$$ (12)

where Ω corresponds to the position of the nuclear membrane, *u*_*ext*_ is the external dye concentration, and *h*_*m*_ is an effective mass transfer coefficient through the boundary Ω. Unlike other parameters that appear in Eqs (9)–(11), the value of *h*_*m*_ is difficult to estimate since it depends on multiple electrostatic and other chemical properties of the cytosol and cell membranes, such as macromolecular obstructions to diffusion, partition coefficient, dielectric properties, and specific transporter kinetics. Instead, we can attempt to determine the value of the coefficient *h*_*m*_ by fitting experimental data to Eqs (9)–(12).

Note that under the assumption that Eqs (9)–(12) correctly describe dye incorporation kinetics, the variability among individual cells is driven by the effective mass transfer coefficient *h*_*m*_ and nuclear radius *R*. Indeed, all cells are exposed to an identical dye concentration in cell culture (even if that concentration is itself time-dependent), and all cells (in the same cell cycle phase) have a similar number of available binding sites.

Upon further consideration, one realizes that the model described by Eqs (9)–(11) is inadequate. Dynamics and steady-state prediction based on Eqs (9)–(11) cannot adequately explain the experimental data (cf. S1 Text). Briefly, in the steady-state, the free extracellular dye concentration will be the same as the intracellular concentration, *u*_*ext*_ = *u*_*st*_. Therefore, the bound dye concentration in the steady-state is completely insensitive to *u*_*ext*_ in the range of concentrations higher than *K*_*d*_ ∼ 0.01 *μM*; however, we observed a sensitivity to dye concentration in cell culture in the concentration range of 0.1 *μM* − 10 *μM*. We note that with the introduction of continuous extracellular dye depletion through the boundary condition, Eq (12) does not remedy the inadequacy of the model described by Eqs (9)–(11) (cf. S1 Text for details). Other model modifications are, therefore, required to account for the observed experimental data.

A local dye concentration in excess of the *K*_*d*_ is a principal reason for the failure of the simple passive diffusion model of Eqs (9)–(11) to recapitulate the observed experimental data. The introduction of a barrier (such as a limiting membrane compartment) results in slower kinetics, as we have seen for small values of the Biot number (dimensionless transfer coefficient, $B_{i}=\frac{h_{m}\;R_{n}}{D}$) (cf. S1 Text), but by itself does not lead to a reduction in the local free dye concentration at later time points. This reduction in free dye concentration could be achieved by active transport of the dye molecules through the cell membrane boundary; however, we observed that cell fixation with formaldehyde does not qualitatively change the fluorescence kinetics. Thus, we turned to other possible explanations for the experimental observations, chief among which is non-specific binding leading to apparent anomalous diffusion.

### Buffering by non-specific binding and anomalous diffusion

Another possible explanation for the reduction in free dye concentration is ‘buffering’ by non-specific (i.e., weaker) binding to other macromolecules in the cytoplasm and nucleus. One obvious suspect in this regard is DNA itself, since dye binding to different base pair sequences occurs and results in much lower or undetectable fluorescence.

If such nonspecific binding (low affinity, high capacity) is a correct explanation for the significant reduction in free dye concentration inside the nucleus, one would expect much higher uptake of the dye by the cells during the course of the experiment than expected from specific binding alone. Indeed, if the dye binds only to the specific high affinity sites that constitute a small fraction (∼ 1%) of total DNA, the effect of dye binding to these specific sites on total dye concentration is expected to be small. In our experimental setting, the number of cells per well is ∼ 5 ⋅ 10^4^, and, therefore, the number of total base pairs per well bp_tot_ is ∼ 1.5 ⋅ 10^14^. This number can serve as the basis for a rough estimate of the number of non-specific binding sites. For a typical dye concentration of 1 *μM* in a cell culture well of 150 *μl* volume, the number of available dye molecules dye_tot_ is ∼ 9 ⋅ 10^13^, which is comparable to bp_tot_. Provided that only ∼ 1% of total DNA binds dye molecules specifically [11], depletion of the total dye pool should be negligible with exclusive specific binding. We, however, observed a significant depletion of dye not only at [dye] = 1 *μM*, but also at higher dye concentrations (*vide infra*). This finding is consistent with lower affinity binding of high capacity.

The amount of non-specific binding sites that act as a dye buffer is proportional to cell density. In order to quantitate this relationship accurately, we used suspended fixed cells, which allows one to quantitate this relationship accurately and also to monitor the remaining free dye concentration in cell culture over time. The latter measurement was obtained by cell centrifugation and subsequent analysis of dye in the cell-free supernatant. Subtracting residual dye concentration from the initial concentration, we can estimate the amount of dye taken up by the cells and compare it to the amount of DNA in the cells.

In order to assess different fluorescence conditions, we incubated different combinations of dye concentration and cell density. The resulting fluorescence intensity at late time point (18 hours of dye incubation) is shown in Figures Ga and Gb in S1 Text, where we compare the fluorescence intensity from intact fixed HeLa cells (Fig. Ga) and the extrapolated signal from the calf thymus DNA (CT) titration data set (Fig. Gb). Namely, we extrapolated a CT signal assuming 6 *pg*/*cell* DNA concentration using CT/dye titration data shown in Figure H in S1 Text. Note that high dye concentration leads to quenching of the fluorescence signal (see also [11]) in CT, for which reason we restricted our analysis to dye concentrations < 8 *μg*/*ml*.

The results of Figures Ga and Gb suggest there may exist dye binding molecules in addition to the specific binding sites in the minor groove of DNA (e.g., other DNA binding sies, RNA, and/or proteins) that would account for higher fluorescence intensity in cells compared to cell-free CT standards. The existence of buffering molecules would also explain less tight binding that manifests in significantly more gradual titration curves for cells compared to cell-free CT samples.

We estimated residual (free) dye concentration in cell suspension samples using the standard CT method (N.B., we could not measure free dye by simple light absorption owing to sensitivity limits). Cells were centrifuged at 8000 *g*, and the collected supernatant was incubated with a fixed concentration of CT (≈ 100 *μg*/*ml*). The CT standard was obtained by titrating various dye concentrations in the presence of the same concentration of CT as above (cf. Figure I in S1 Text). Using this approach, one can estimate the residual free dye concentration in the cell suspensions. The results are shown in Figures Gc and Gd for the corresponding raw data (Fig. Gc) and extrapolated values of free dye in supernatant samples (Fig. Gd). Owing to the second incubation step that is necessary in this approach, the original free dye was diluted two-fold, which was taken into account in the results in Fig. Gd.

We note that due to limited sensitivity of the assay, the free dye concentration could not be accurately assessed for values < 1 *μg*/*ml*. For this reason, we did not apply extrapolation to samples with initial dye concentrations less than 4 *μg*/*ml*. For high initial dye concentrations, we observed dye uptake that cannot be explained by specific DNA binding alone. Indeed, for a cell density of 2.5 ⋅ 10^5^ *cells*/*ml*, there is approximately 1.5 *μg* of DNA per *ml* volume in solution. The dye uptake by the cells shown in Figure Gd is at least 3 times greater than the total DNA concentration, 4.5 *μg*/*ml* for [*dye*] = 8 *μg*/*ml*. Taking into account that only a fraction of DNA is available for specific binding (cf. CT titration data, Fig. H in S1 Text), there must exist (macro)molecules with low binding affinity and much higher concentration (capacity) than specific DNA sites to account for the magnitude of dye uptake we observed.

### Dynamics and steady-state solution of non-specific binding model: The basis for anomalous diffusion

Before we turn to a numerical simulation of the model that takes into account non-specific binding interactions, let us demonstrate the resulting behavior in a single cell. It is intuitively clear that *any* binding and dissociation reactions, whether specific or non-specific, can lead to anomalous diffusion of molecule(s) in the cell by impairing the theoretical unimpeded diffusion of the molecule in the cytosol. (Anomalous diffusion has been studied in some limiting cases of these interactions under the rubrics of ‘excluded volume’ and ‘fractal structure of the cell’; for review see e.g., [12, 13]).

We demonstrate anomalous diffusion behavior for a ‘toy’ system: diffusion of a single particle (drug molecule) in bulk. In what follows, we assume that the particle undergoes a random walk on a *d*-dimensional lattice and can interact with particles uniformly embedded in nodes of the lattice. A diffusing particle can be in one of two possible probabilistic states, *p* and *q* (i.e., free or bound, respectively). We introduce transition rates *k*_+_ and *k*_−_ between these two states (which are the microscopic analogues to *k*_on_ and *k*_off_, respectively). The major advantage of the model compared to a general case is linearity and, hence, the existence of an exact solution. Indeed, the continuous version of this model yields:

$$\begin{array}{c} R_{1}\left(p,q\right)=k_{+}\;p\left(x,t\right)-k_{-}\;q\left(x,t\right) \end{array}$$ (13)

$$\begin{array}{c} \partial_{t}p\left(x,t\right)=D\;\nabla_{x}^{2}p-R_{1}\left(p,q\right) \end{array}$$ (14)

$$\begin{array}{c} \partial_{t}q\left(x,t\right)=R_{1}\left(p,q\right) \end{array}$$ (15)

subject to boundary and initial conditions:

$$\begin{array}{c} p(\Omega ,t)=0 \end{array}$$ (16)

$$\begin{array}{c} p(x,0)=\delta (x) \end{array}$$ (17)

$$\begin{array}{c} q(x,0)=0 \end{array}$$ (18)

where the boundary Ω is assumed to be very far from the origin, *x* = 0.

In this setting, we wish to calculate the mean square displacement 〈*x*^2^〉 of the particle from its origin:

$$\begin{array}{c} \langle x^{2}\rangle =\int dx\;x^{2}\;\left(p+q\right) \end{array}$$ (19)

On very short time scales *k*_+_ *t* ≪ 1, the diffusion is normal and is described by the usual rate law 〈*x*^2^〉 = 2 *dDt* where *d* is the lattice dimensionality. In the long time regime (*k*_+_ + *k*_−_)*t* ≫ 1, one expects the following asymptotic behavior:

$$\begin{array}{c} \langle x^{2}\rangle ≃2\;d\;D^{*}\;t \end{array}$$ (20)

$$\begin{array}{c} D^{*}=D\frac{k_{-}}{k_{-}+k_{+}} \end{array}$$ (21)

The asymptotic behavior Eqs (20) and (21) is due to translational symmetry, namely, diffusion and reaction processes do not depend on the position of the particle on the lattice. Indeed, since the particle can move only while in a free state, the late time asymptotic diffusion rate is proportional to the steady-state probability that the particle is free at any given time. In order to derive an exact solution to the mean square displacement in the case of the toy model Eqs (13)–(18), we first derive the relaxation dynamics of the free particle state *p*_0_(*t*) in the case of *d* = 0 (single site lattice, corresponds to *D* = 0 in Eqs (13)–(15)):

$$\begin{array}{c} p_{0}\left(t\right)=\frac{k_{-}}{k_{-}+k_{+}}+\frac{k_{+}}{k_{-}+k_{+}}e^{-(k_{-}+k_{+})t} \end{array}$$ (22)

The exact solution of Eqs (13)–(15) is, therefore, given by:

$$\begin{array}{c} \langle x^{2}\rangle =2\;d\;D\int_{0}^{t}d\tau \;p_{0}\left(\tau \right) \end{array}$$ (23)

$$\begin{array}{c} \langle x^{2}\rangle =\delta [1-e^{-(k_{-}+k_{+})t}]+2\;d\;D^{*}\;t \end{array}$$ (24)

$$\begin{array}{c} \delta =2\;d\;D\frac{k_{+}}{{\left(k_{-}+k_{+}\right)}^{2}} \end{array}$$ (25)

**Fig 3:**
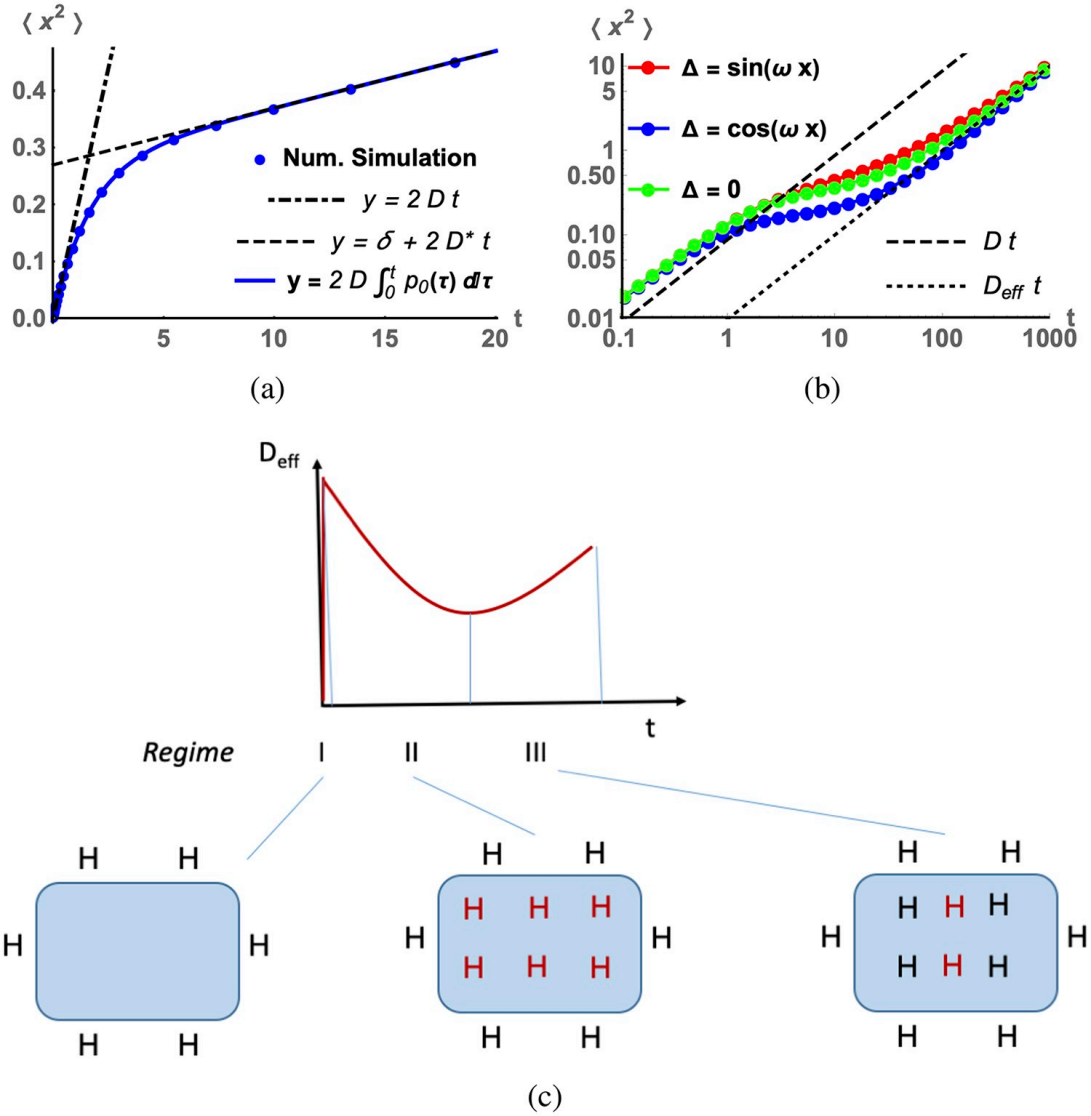
(a) The one-dimensional simulation of the toy model, Eqs (13)–(15). Parameter values are *D* = 0.088, *k*_−_ = 0.035, *k*_+_ = 0.58 (uniform). Offset *δ* has been determined from Eq (25): *δ* ≈ 0.27. (b) Log-log plot of mean square displacement for non-uniform distribution of binding rate, $k_{+}=k_{+}^{0}+\Delta \left(x\right)$. Parameter values are the same as in (a), and perturbation Δ(*x*) is periodic with frequency $\omega =\frac{2\pi }{5}$. (c) Conceptual diagram of effective diffusion rate as a function of time for the full problem. Black H is dye bound to specific DNA binding sites and red H is dye bound to less specific, high capacity, low affinity DNA binding sites.

Here, the integral over time in Eq (23) corresponds to the total time the diffusing particle remains in the free state during observation time *t*. The numerical simulation of the mean square displacement for Eqs (13)–(15) in 1 *d* is presented in Fig 3a along with the exact solution, Eqs (23)–(25).

The mean square displacement in the presence of association and dissociation reactions for our model exhibits anomalous diffusion in the transient time regime, Eqs (23)–(25). One expects that if interacting particles are embedded on the lattice in a non-uniform fashion, this pattern will persist much longer since under these conditions a diffusing particle will explore different regions of space, and microscopic reaction rates *k*_+_ and *k*_−_ will become position-dependent.

We next considered a locally non-uniform distribution of interacting particles (which is the case for DNA binding sites) and compared the time dependence of 〈*x*^2^〉 to the case of a uniform distribution (with identical average *k*_+_ values in both cases). One may expect that after sufficient space exploration time (late time limit), 〈*x*^2^〉 would exhibit similar asymptotic behavior for both uniform and non-uniform local distributions of interacting particles. In order to demonstrate this fact, we introduced a local perturbation to the binding rate, $k_{+}=k_{+}^{0}+\Delta \left(x\right)$. For periodic local perturbation, Δ ∝ sin(*ωx* + *θ*), simulated mean square displacements 〈*x*^2^〉 are shown in Fig 3b.

Even for uniformly distributed interacting particles, the diffusion is anomalous if more than a single particle performs a random walk on the lattice. This anomalous diffusion occurs because binding and dissociation rates become time-dependent. Indeed, for a given walker, the state of interacting particles at any site on the lattice depends on whether other walkers are engaged at that site.

Disregarding spatial fluctuations, we can formulate a mean-field approximation for the multiple walkers problem by substituting *k*_+_ ∼ *k*_*on*_ *ρ*_*f*_(*t*), where *ρ*_*f*_(*t*) describes the time-dependent concentration of available (i.e., not bound) reactive species interacting with the walker. For low dye (walker) concentrations, we can estimate the effective diffusion rate using Eqs (20) and (21):

$$\begin{array}{c} D^{*}=D\frac{k_{off}}{k_{off}+k_{on}\rho } \end{array}$$ (26)

$$\begin{array}{c} D^{*}=D\frac{K_{d}}{K_{d}+\rho } \end{array}$$ (27)

where it is assumed that the free reactive species concentration does not change significantly, *ρ*_*f*_(*t*) ≈ *ρ*. For the sub-micromolar dissociation constant *K*_*d*_ of non-specific binding reported in [11] and high *intracellular* concentrations with lower affinity binding sites *ρ* ≥ 100 *μM*, one may expect a 10^2^ − 10^3^ times slower effective diffusion rate D* compared to the diffusion of dye in water, *D*. This slow effective diffusion constant is consistent with the time scale we observed in our experiments. We note here that the mechanism of retardation of dye transport through membrane(s) most likely is also driven by non-specific interaction between dye and lipid molecules or dye and (membrane) protein molecules present in high local concentration.

Using Eq (27) we can approximate the time-dependent changes in effective diffusion constant in the bulk phase by assuming

$$\begin{array}{c} D_{eff}\left(t\right)=D\frac{K_{d}}{K_{d}+\rho \left(t\right)} \end{array}$$ (28)

where *ρ*(*t*) is a time-dependent spatial average concentration of available binding sites. This is, of course, a crude approximation that completely ignores spatial fluctuations in interacting particle density. In order to estimate the time dependence of *ρ*(*t*), we (i) assume that all cells are identical, and (ii) once again ignore spatial fluctuations in the distribution of interacting particles. Under these assumptions, we derive the autonomous evolution equation for *ρ*(*t*):

$$\begin{array}{c} \frac{d}{dt}\;\rho =-k_{on}\;\left(u_{0}+\rho -\rho_{tot}\right)\;\rho +k_{off}\;\left(\rho_{tot}-\rho \right) \end{array}$$ (29)

$$\begin{array}{c} \rho \left(0\right)=\rho_{tot} \end{array}$$ (30)

where we define *ρ*_*tot*_ as a total amount (capacity) of DNA and *u*_0_ is an initial amount of dye available for each cell. The solution of the nonlinear equation, Eqs (29) and (30), is:

$$\begin{array}{c} \rho \left(t\right)=\rho_{1}+\rho_{2}\tanh(\frac{1}{2}\beta \;t+\rho_{3}) \end{array}$$ (31)

$$\begin{array}{c} \beta =\sqrt{{\left[k_{off}+k_{on}\;u_{0}+k_{on}\;\rho_{0}\right]}^{2}-4\;\left[k_{on}\;u_{0}\right]\;\left[k_{on}\;\rho_{0}\right]} \end{array}$$ (32)

where all parameters, *ρ*_1_, *ρ*_2_, *ρ*_3_, and *β*, depend on reaction rates and initial conditions.

In order to estimate the value of rate *β*, we consider a case wherein *u*_0_ ≈ *ρ*_0_ ∼ 1 *μM*. (Note that here *ρ*_0_ corresponds to the average concentration of DNA in culture media, not in the individual cell). In this case one derives:

$$\begin{array}{c} \beta ∼2\;k_{off}\;\sqrt{\rho_{0}/K_{d}} \end{array}$$ (33)

Experimentally, we observed a very slow effective dissociation rate *k_off_* ≲ 10^−5^ *sec*^−1^, (see Fig. B in S1 Text). Hence, the dye depletion rate can be approximated from the above as *β* ≲ 10^−4^ *sec*^−1^ for sub-millimolar non-specific dissociation constant *K*_*d*_.

The derivation of the solution Eqs (31) and (32) and its generalization to the case of multiple binding species can be found in S1 Text; also note Figure Ja for a comparison of analytical and numerical solutions for this case (cf. S1 Text section, Mean-field Solution to Autonomous Binary Reaction Model).

We also used a numerical simulations scheme that allows us to trace a single “molecule” (walker) displacement during a stochastic reaction-diffusion process implemented in 3d space. The typical time traces of 〈*x*^2^〉 for mobile species in the absence and presence of interactions with stationary interacting species are shown in Figs. Jc-Je in S1 Text. A conceptual diagram of the time dependence of $\tilde{D}$ is shown in Fig 3c.

In order to incorporate nonspecific binding in the model defined by Eqs (9)–(11), we introduced an additional term that corresponds to an average (lower affinity, relatively) non-specific binding site. We further assume that this non-specific binding site is immobile compared to free dye in the time course of the experiment:

$$\begin{array}{c} R\left(u,v\right)=k_{\text{on}}\;u\;\left(c-v\right)-k_{\text{off}}\;v \end{array}$$ (34)

$$\begin{array}{c} R^{n}\left(u,v^{n}\right)=k_{\text{on}}^{n}\;u\;\left(c^{n}-v^{n}\right)-k_{\text{off}}^{n}\;v^{n} \end{array}$$ (35)

$$\begin{array}{c} \partial_{t}u\left(x,t\right)=D\;\nabla_{x}^{2}u-R\left(u,v\right)-R^{n}\left(u,v^{n}\right) \end{array}$$ (36)

$$\begin{array}{c} \partial_{t}v\left(x,t\right)=R\left(u,v\right) \end{array}$$ (37)

$$\begin{array}{c} \partial_{t}v^{n}\left(x,t\right)=R^{n}\left(u,v^{n}\right) \end{array}$$ (38)

**Fig 4:**
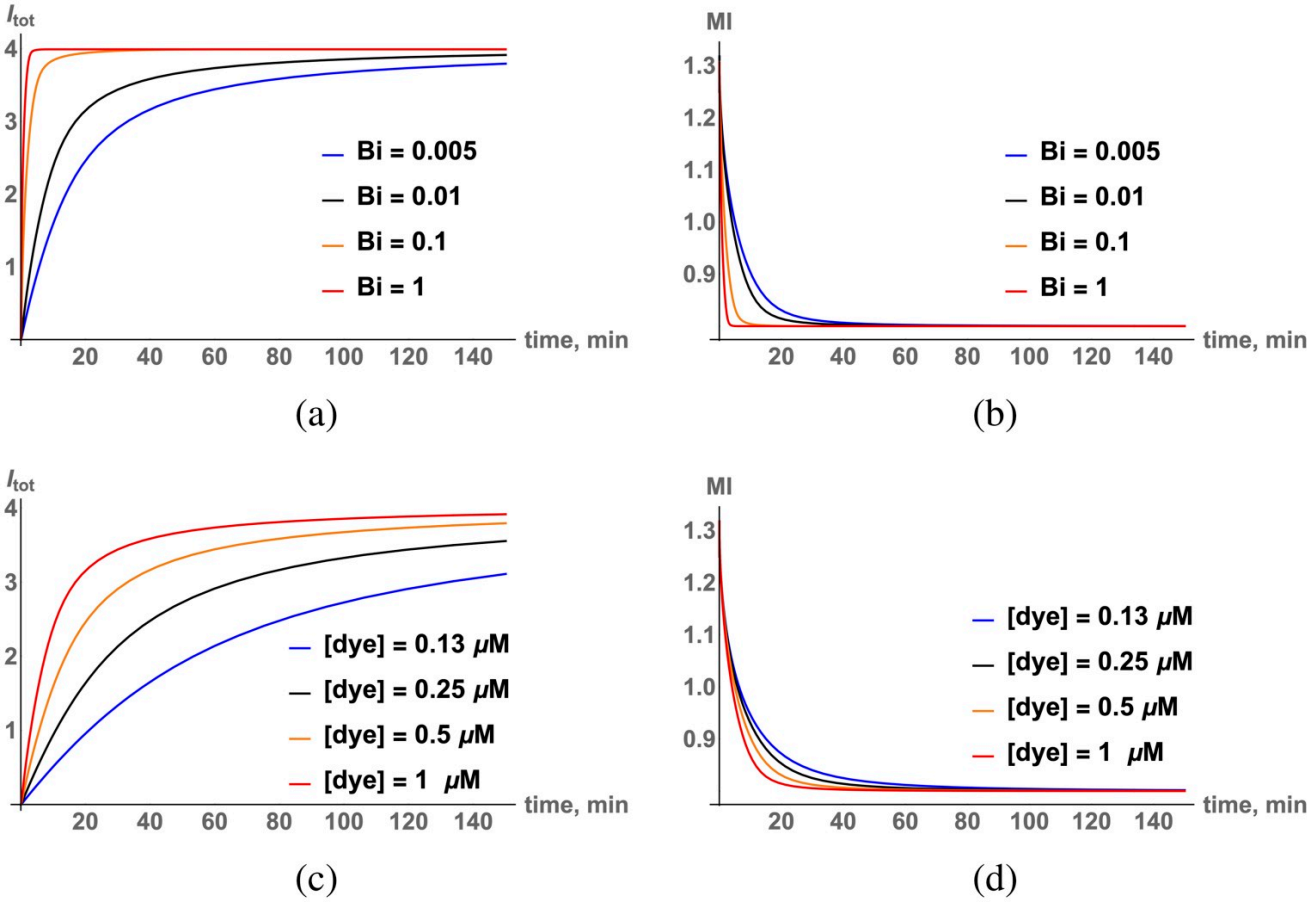
(a) Numerical simulation (time dependent external concentration *u*_*ext*_(*t*)) of overall dye incorporation per unit volume for different Biot numbers, *u*_*ext*_ = 1 *μM*. (b) Moment of inertia *M*_2_ as a function of time, same conditions as in (a). (c) Numerical simulation of overall dye incorporation per unit volume for different [dye], *u*_*ext*_ = 1 *μM*. (d) Moment of inertia *M*_2_ as a function of time, same conditions as in (c).

Here, the superscript *ns* refers to a (generic) non-specific binding site. We include an estimate of two additional parameters in the model in Eqs (34)–(38), $k_{\text{on}}^{n}$ and $k_{\text{off}}^{n}$, from reference [7] and assume that the concentration of non-specific binding sites is ∼ 100-fold greater than specific sites, i.e., *c*^*n*^ ∼ 100*c*. The results of numerical simulation of the model described by Eqs (34)–(38) are shown in Fig 4a–4d.

**Fig 5:**
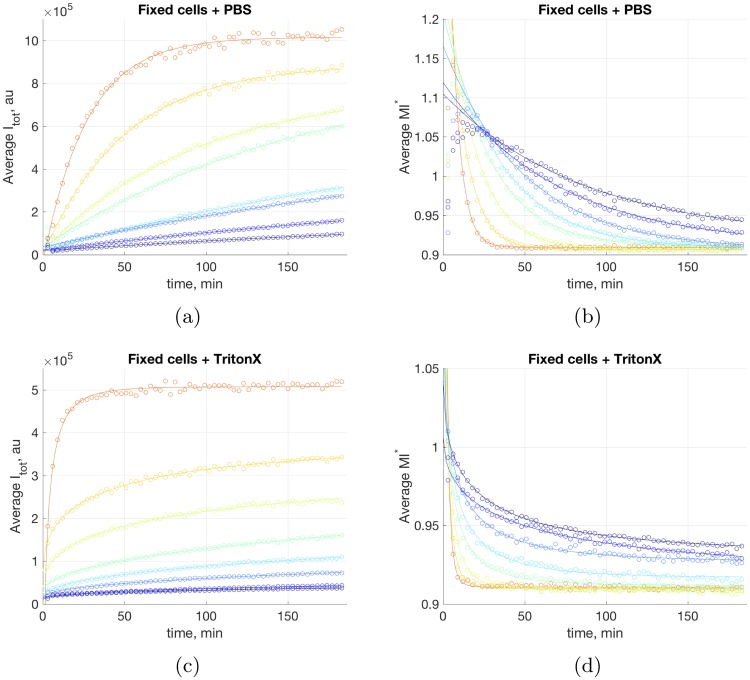
(a) *I*_*tot*_ and (b) moment of inertia *M*_2_, for different dye concentrations, population average, fixed cells. (c) *I*_*tot*_ and (d) moment of inertia *M*_2_, for different dye concentrations, Triton X-100-treated fixed cells, conditions as in (a) and (b).

The corresponding experimental results are shown in Fig 5a and 5b. Numerical simulations of the full non-specific interaction model support the prediction of the qualitative estimate above that both dye incorporation and front propagation are consistent with a slow diffusion process. The front dynamics is not described by a simple exponent as expected in the case of normal diffusion but, rather, consistent with the anomalous behavior discussed above.

### Single cell heterogeneity in drug incorporation

We turn next to the heterogeneity in incorporation kinetics. To begin, we examine the steady-state levels of dye incorporation. A typical histogram of steady-state fluorescence intensity is presented in Figure L in S1 Text. This multi-modal distribution is likely to be driven by the cell cycle, with the two largest peaks corresponding to *G*_1_ and *G*_2_ phases. The coefficient of variation (*CV*) for cells in *G*_1_ and *G*_2_ states is similar and has typical values of 0.1, excluding outliers (e.g., segmentation errors in quantifying nuclear fluorescence). We observed that low dye concentrations result in very slow kinetics (cf. Fig 1b) for dynamics of the population average. This slow kinetics is difficult to study experimentally, especially with live cells (tracking individual cells becomes difficult with cell motion over long times). In order to explore a possible link between the variability in kinetics and DNA target state (such as cell cycle phase), we performed a timed double (sequential) addition experiment, viz., dye was added to cell culture in two sequential steps. If, at the first step of the experiment, the dye concentration is low and it is experimentally impossible to achieve the steady-state, adding high dye concentrations to the cells in the second step allows us to achieve steady-state equilibration. Even though conditions at final equilibrium are different from conditions after the first addition of dye, the DNA binding capacity can be resolved using this method.

Using these sequential addition experimental data, it is straightforward to confirm the existence of the buffering molecules discussed above. If the dye were not depleted from cell culture, one would expect that adding less dye in the second phase of the experiment would result in a decrease or, in the best case, no change in final fluorescence intensity. This is not the case, as shown in Figures Ma and Mb in S1 Text. As an example, consider changes in the average fluorescence intensity for the experimental conditions [dye_1_] = 0.25 *μM*, [dye_2_] = 0.12 *μM* shown in Figure Ma (brown curve). Despite adding a lower concentration of dye, the average fluorescence intensity increases. This behavior persists for higher dye concentrations. For example, the experimental conditions [dye_1_] = [dye_2_] = 1 *μM* also result in an increase of fluorescence intensity (Fig. Mb, red line).

The steady-state dependence of mean and *CV* of individual nuclear intensities on dye concentration are shown in Figure N in S1 Text. We classified cells into two cycle phases based on the final fluorescence intensity observed in the sequential addition experiment. While steady-state intensities display significant variation for different fluorescence conditions (Figures Na and Nb), their degree of variability (*CV*) remains roughly constant for a broad range of dye concentrations (Figures Nc and Nd).

**Fig 6:**
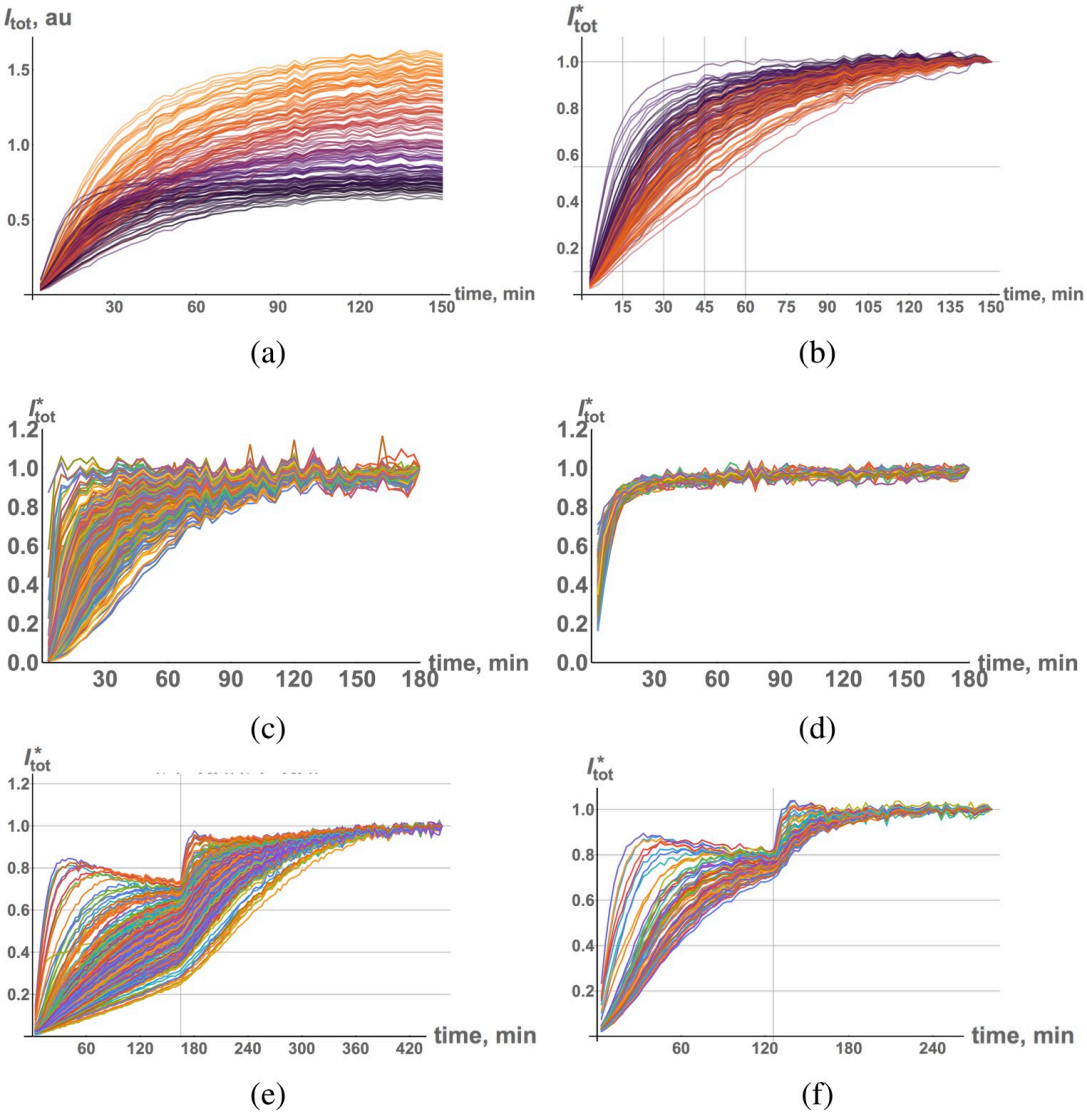
(a) Time traces of overall dye incorporation ([dye] = 1 *μM*) of individual live cells, color corresponds to final intensity. (b) Normalized intensity, $I_{tot}^{*}$, colored as in (a). Traces of normalized intensity, $I_{tot}^{*}$, [dye] = 1 *μM*, (c) fixed cells + PBS; (d) fixed cells + Triton X-100. Traces of individual cell normalized fluorescence intensity, $I_{tot}^{*}$, for the following conditions in the double incubation protocol: (e) [dye_1_] = 0.5 *μM*, [dye_2_] = 0.5 *μM*, and (f) [dye_1_] = 0.5 *μM*, [dye_2_] = 0.25 *μM*.

In contrast to the narrow distribution of dye incorporation in the steady-state, relaxation kinetics toward equilibrium exhibit a much greater variance. To demonstrate this point graphically, we introduce the normalized time-dependent variable $I_{tot}^{*}$ defined as:

$$\begin{array}{c} I_{tot}^{*}=\frac{I_{tot}\left(t\right)}{I_{tot}\left(T\right)}, \end{array}$$ (39)

where *T* is a final dye incubation time point. Time traces of live cells’ raw intensity *I*_*tot*_ and normalized intensity $I_{tot}^{*}$ are shown in Fig 6a and 6b, respectively. The estimated half-life of relaxation ranges from the fastest relaxation rate, *τ*_1/2_ ≈ 10 min, to the slowest relaxation rate, *τ*_1/2_ > 60 (min), for [dye] = 1 *μM*, a 6-fold difference.

It is clear from Fig 6b that the relaxation rate of incorporation correlates with the cell cycle, namely, cells in *G*_1_ phase achieve equilibrium faster than those in *G*_2_ phase. Therefore, the variability in relaxation rates is actually smaller if one takes into account cell cycle state. Even allowing for this distinction, the variability in relaxation rate is still several fold higher (*CV* ∼ 0.6) than the variability in the steady-state fluorescence intensity (*CV* ∼ 0.15) (Figs. Nc, Nd, Oa and Ob in S1 Text).

### Membrane components determine variability in drug kinetics

In order to determine the factors controlling the variability in dye kinetics, we performed experiments on fixed cells with permeabilized membranes (using Triton X-100). The resulting kinetics is depicted in Fig 6c and 6d. Cells permeabilized with Triton X-100 display fluorescence dynamics that is *initially* significantly faster (about 3-fold on average) than intact cells, consistent with the microplate reader data discussed above for the HeLa cell line, (cf. Fig 5a and 5c). The variability in intensity of permeabilized cells appears significantly lower compared to that of intact cells. As a result, late time behavior becomes almost uniform for permeabilized cells.

In addition and importantly, the data from the sequential addition experiments show that variability in kinetics among cells persists after the first addition using non-permeabilized fixed cells (Fig 6e and 6f). Therefore, the factor(s) that cause variability do not “saturate” during the incubation phase. Since the interaction of the dye with membrane(s) is most likely driven by non-specific association/dissociation reactions, one would expect that saturation of binding sites would result in more uniform dynamics during the second addition phase of the experiment. This result suggests that there may exist factor(s) other than transport through the cell membrane that control(s) variability in incorporation kinetics.

Some of the fixed cells’ time traces exhibit other interesting behaviors. Namely, a few traces reach peak fluorescence intensity during the incubation period after which their fluorescence intensity decreases with time. This biphasic behavior is especially apparent for low dye concentrations (Fig 6e and 6f). We examined images of cells that exhibit this behavior and discovered that the nuclei of these cells have region(s) that incorporate dye very quickly compared to the rest of the nucleus. The very same region is responsible for a decrease in fluorescence signal after it peaks. We hypothesized that the regions with fast reaction kinetics correspond to micro-damaged areas of the nucleus (i.e, exposed/accessible DNA binding sites) owing to fixation. The effective diffusion and, hence, mixing, of the dye is, therefore, enhanced. Under this assumption, the peak fluorescence intensity is caused by a decrease in the extracellular dye concentration during the time course of the experiment (due to depletion of the free dye by cells discussed above). This observation supports the hypothesis that the reason for accelerated kinetics in the presence of detergents might not only be a consequence of membrane dissolution, but also of the presence of other binding species and compartments within the cell.

### Doxorubicin binding and implications for pharmacotherapy

We next investigated whether anomalous and slow diffusion in cells is unique to Hoechst dye. To this end, we studied the incorporation dynamics of another DNA binding drug, doxorubicin, a potent cancer chemotherapeutic agent. In order to characterize doxorubicin incorporation, we employed an indirect method based on doxorubicin-DNA intercalation competition with Hoechst dye 33342 [14, 15]. Since the total pool of DNA sites specific for binding to doxorubicin and Hoechst dye is limited, one may expect that dye fluorescence in cells would depend on the local concentration of doxorubicin.

**Fig 7:**
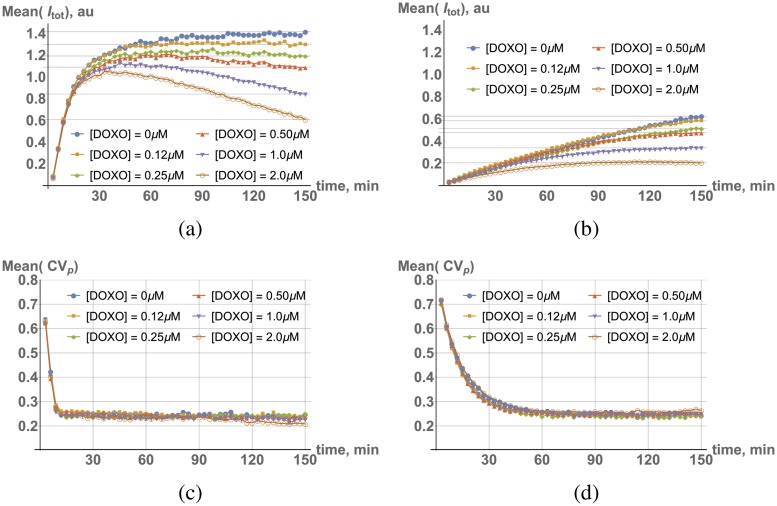
Population average *I*_*tot*_ time dependences for different doxorubicin concentrations in live cell culture, (a) [dye] = 8 *μM* and (b) [dye] = 0.25 *μM*. (c) and (d): Population average *CV*_*p*_, same conditions as above.

We, indeed, observed this antagonistic (competitive) effect at the single cell level. If doxorubicin is delivered at the same time or later than dye to cultured cells, we observed a peak pattern in time traces shown in Fig 7a and 7b.

The peak position corresponds to the point at which doxorubicin concentration in the nucleus becomes high enough to compete effectively with bound dye for specific DNA binding sites. The timing of the peak fluorescence depends on relative dye and doxorubicin concentrations in cell culture, as can be seen in the case of high or low dye concentrations shown in Fig 7a and 7b, respectively. (A similar pattern is observed in fixed cells, Figs. Qa and Qb in S1 Text).

If cells are pre-treated with doxorubicin several hours prior to dye addition, however, traces exhibit simple plateau saturation (which is [*Dox*]-dependent). This observation leads to the conclusion that it takes a fairly long period of time for doxorubicin to achieve sufficient intracellular concentrations to compete effectively with Hoechst dye. As in the dye case, this time period is [*Dox*]-dependent (see timing of peaks in Fig 7a). Thus, slow incorporation is most likely a common feature of DNA binding drugs for exactly the same reasons as for Hoechst dye: (i) high local DNA concentrations, and (ii) non-specific interactions with other macromolecules in cells. Since these factors affect both dye and doxorubicin molecules similarly, one may expect that the kinetics of dye incorporation can be used as a proxy for doxorubicin kinetics.

Surprisingly, dye homogenization in cells does not seem to be affected by co-incubation with doxorubicin. This conclusion is supported by the time traces of either moment of inertia *M*_2_ introduced above or another proxy for homogenization, the coefficient of variation in individual nuclear pixel intensities *CV*_*p*_. The observed dynamics of *CV*_*p*_ is shown in Fig 7c and 7d, and unlike total intensity of incorporation (Fig 7a and 7b), is largely [Dox]-independent. (A similar pattern is seen in fixed cells, Figs. Qc and Qd in S1 Text). The most likely explanation for this behavior is the very similar effective diffusion properties of dye and doxorubicin, since one would otherwise expect non-uniform displacement of bound dye molecules throughout the nucleus.

**Fig 8:**
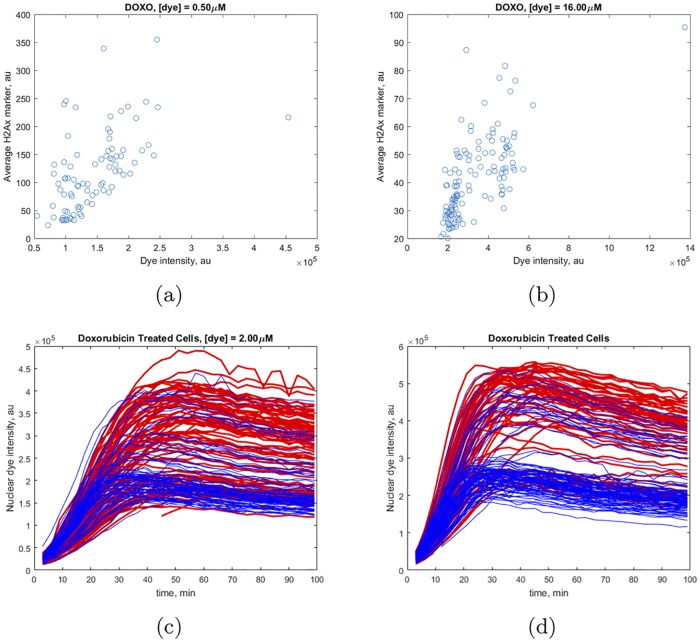
Extent of DNA damage due to doxorubicin treatment vs. Hoechst dye incorporation. Cells were incubated with [*Dox*] = 1 *μM* and (a) [*dye*] = 0.5 *μM* or (b) [*dye*] = 16 *μM* correspondingly. Time traces of individual cells incubated with [*Dox*] = 1 *μM* and (c) [*dye*] = 2 *μM* or (d) [*dye*] = 4 *μM*. Blue colored traces correspond to a lower extent of DNA damage as determined using the marker *γ*-H2Ax. Red colored traces correspond to a higher extent of DNA damage.

Doxorubicin is, of course, a clinically used chemotherapeutic agent and, hence, one can quantify drug efficacy in individual cells by assessing the time course of DNA damage after incubation. We used *γ*-H2Ax antibody intensity as a proxy for DNA damage in cells. To simplify phenotype characterization, we dichotomized DNA damage by introducing an assay threshold. The threshold was set based on a comparison of *γ*-H2Ax antibody intensity in doxorubicin-treated and untreated conditions. First, we observed that dye acts as a buffer at high dye concentration by competing for binding with doxorubicin in the DNA minor groove (Fig 8a and 8b).

For high dye concentration (16 *μM*), the extent of DNA damage is below the threshold (corresponding to an intensity of 100 arbitrary units of *γ*-H2Ax antibody) for most cells. By contrast, incubation with low dye concentration (0.5 *μM*) leads to extensive DNA damage for a large fraction of cells. This result is consistent with the peak pattern for dye and doxorubicin co-incubation discussed above, which is also driven by competition for DNA binding. In addition, slow dynamics of drug incorporation leads to a higher extent of DNA damage, which is a non-trivial effect. To demonstrate this phenomenon, we plotted time traces of dye fluorescence intensity in individual cells treated with doxorubicin, as depicted in Fig 8c and 8d. Most of the cells that undergo DNA damage are in *G*_2_ phase, which is typically characterized by slower incorporation kinetics compared to cells in *G*_1_ phase; however, cells that exhibit a lesser degree of DNA damage in *G*_2_ phase typically achieve peak dye fluorescence intensity faster. The temporal position of the peak is related to the rate of intracellular doxorubicin accumulation. Hence, counterintuitively, cells are more likely to escape DNA damage if doxorubicin incorporation dynamics is rapid.

## Discussion

We observed several striking features of binding kinetics in our model system: First, both binding and dissociation of dye are much slower (by three orders of magnitude) in cells than in cell-free systems. In fact, the effective dissociation rate is so slow that binding is essentially irreversible. We show that this dye “trapping” in the nucleus is due to (i) high local DNA concentrations; (ii) higher capacity, lower affinity interactions with other macromolecules; and (iii) lipid membrane(s) partitioning and permeability characteristics. Second, we observed reaction front propagation by monitoring the spatial distribution of the dye in the nucleus over time. Temporal dynamics of front propagation is also slow compared to the dye diffusion rate in water, and is most likely controlled by the same factors as mentioned above. Third, slow drug intake/extrusion is not unique to the dye. We demonstrate that a clinically used drug (doxorubicin) that has a binding mechanism similar to the Hoechst dye also exhibits slow binding kinetics. Finally, we demonstrate that drug incorporation dynamics varies significantly among individual cells. On the characteristic time scales of our experiments (minutes to hours), some of the heterogeneity is due to the effects of the cell membrane compartments in the cell and their kinetic effects on dye entry into the cytosol and nucleus. We observed a correlation between the dynamics of drug incorporation and its efficacy in causing DNA damage using doxorubicin as a drug and dye dynamics as a proxy for the kinetic properties of individual cells.

Effectively irreversible binding has a very interesting implication in terms of distribution of incorporated drug between cells. For sub- or even micromolar drug concentrations, one expects that cells with fast incorporation kinetics would effectively serve as a sink reducing drug availability to cells with slower kinetics. This behavior might be interpreted as “passive” drug resistance in subpopulations of cells. There might be nothing biologically unique about this cell subpopulation; however, the existence of cells that can take up drug rapidly is a driving factor for the drug-resistant subpopulation. The possible clinical solution in this case might be completely counterintuitive. Instead of improving targeting of passively resistant cells, the drug-sensitive subpopulation of resistant cells needs to be treated with reagents that *decrease* their drug incorporation rate. A similar notion of the effective sink might be applicable on a larger spatial scale to cells in solid tumors. Some cells (e.g., those in outer layers) may act as a shield, taking up the drug, which, in turn, may facilitate drug resistance of the inner layers of cells in the tumor.

Non-specific interactions are often short range, driven by chemical reaction requiring close proximity of interacting species. Owing to a crowded intracellular environment, these interactions can effectively trap drug molecules in subcellular regions with high local concentrations of non-specific binders. Hence, non-specific interactions between drug and macromolecules present in the cell may result in slow and anomalous intracellular diffusion of drug molecules. Since the spatial organization of the intracellular micro-environments depends on cell cycle phase, one may expect that drug incorporation kinetics will also be cell cycle-dependent.

The heterogeneity of drug incorporation is not driven exclusively by cell cycle state. We observed a high degree of variability in kinetics for both *G*_1_ and *G*_2_ subpopulations of cells. While active transport has been shown to be an important factor contributing to drug incorporation efficacy on long time scales, we have not detected significant changes in short-term kinetics between live and fixed cells at the population average level (at least not in HeLa and MFC10A cell lines). Hence, other factors, such as relative spatial organization of drug targets and non-specific interacting molecules, likely drive variability in incorporation kinetics and account for anomalous diffusion characterization of the drug.

Slow drug transport through the plasma membrane is often empirically taken into account during drug design and optimization stages. We observed, however, that a slow diffusion process occurs within a cell, as well, at least for cationic DNA-binding small molecules, such as Hoechst dye and doxorubicin. The immediate consequence of this slow diffusion is a dramatic mismatch between kinetic reaction rates *in vivo* and *in vitro*, which we observed experimentally. Hence, we believe that non-specific interactions have to be taken into account in order to describe drug kinetics adequately. By so doing, it is likely that different strategies will be needed to optimize drug efficacy and minimize drug resistance.

## Supporting information

S1 Text Supporting information. (PDF)

S1 File Embedded videos. (PPTX)

## References

[1] Prasanphanich AF, White DE, Gran MA, Kemp ML. Kinetic Modeling of ABCG2 Transporter Heterogeneity: A Quantitative, Single-Cell Analysis of the Side Population Assay. PLoS Comput Biol. 2016;12(11):e1005188 10.1371/journal.pcbi.1005188 27851764 PMC5113006

[2] Miller MA, Gadde S, Pfirschke C, Engblom C, Sprachman MM, Kohler RH, et al Predicting therapeutic nanomedicine efficacy using a companion magnetic resonance imaging nanoparticle. Sci Transl Med. 2015;7(314):314ra183 10.1126/scitranslmed.aac6522 26582898 PMC5462466

[3] Thurber GM, Yang KS, Reiner T, Kohler RH, Sorger P, Mitchison T, et al Single-cell and subcellular pharmacokinetic imaging allows insight into drug action in vivo. Nature Communications. 2013;4(1). 10.1038/ncomms2506 23422672 PMC3579506

[4] Karolak A, Estrella VC, Huynh AS, Chen T, Vagner J, Morse DL, et al Targeting Ligand Specificity Linked to Tumor Tissue Topological Heterogeneity via Single-Cell Micro-Pharmacological Modeling. Scientific Reports. 2018;8(1). 10.1038/s41598-018-21883-z 29483578 PMC5827036

[5] Landau LD, Lifshitz EM. Mechanics, vol. 1 Course of Theoretical Physics. 1976;3.

[6] Hu MK. Visual pattern recognition by moment invariants. IRE Transactions on Information Theory. 1962;8(2):179–187. 10.1109/TIT.1962.1057692

[7] Breusegem SY, Clegg RM, Loontiens FG. Base-sequence specificity of Hoechst 33258 and DAPI binding to five (A/T)4 DNA sites with kinetic evidence for more than one high-affinity Hoechst 33258-AATT complex. J Mol Biol. 2002;315(5):1049–1061. 10.1006/jmbi.2001.5301 11827475

[8] Furusawa H, Nakayama H, Funasaki M, Okahata Y. Kinetic characterization of small DNA-binding molecules interacting with a DNA strand on a quartz crystal microbalance. Anal Biochem. 2016;492:34–42. 10.1016/j.ab.2015.09.015 26408811

[9] Tissera H, Kodiha M, Stochaj U. Nuclear envelopes show cell-type specific sensitivity for the permeabilization with digitonin Protocol Exchange.

[10] Bray D. Cell movements: from molecules to motility Garland Science; 2000.

[11] Loontiens FG, Regenfuss P, Zechel A, Dumortier L, Clegg RM. Binding characteristics of Hoechst 33258 with calf thymus DNA, poly [d (AT)] and d (CCGGAATTCCGG): multiple stoichiometries and determination of tight binding with a wide spectrum of site affinities. Biochemistry. 1990;29(38):9029–9039. 10.1021/bi00490a021 1702995

[12] Golding I, Cox E. Physical nature of bacterial cytoplasm. Phys Rev Lett. 2006;96(9). 10.1103/PhysRevLett.96.098102 16606319

[13] Hoefling F, Franosch T. Anomalous transport in the crowded world of biological cells. Rep Prog Phys. 2013;76(4). 10.1088/0034-4885/76/4/046602 23481518

[14] Hovorka O, Subr V, Větvička D, Kovář L, Strohalm J, Strohalm M, et al Spectral analysis of doxorubicin accumulation and the indirect quantification of its DNA intercalation. Eur J Pharm Biopharm. 2010;76(3):514–524. 10.1016/j.ejpb.2010.07.008 20638475

[15] Calvert RJ, Vohra S. Doxorubicin-treated H9c2 cells: caution with luminescent ATP and Hoechst 33258 assays. In Vitro Cell Dev Biol Anim. 2013;49(2):95–96. 10.1007/s11626-012-9573-1 23288412

