# Supplementary material for: Determinants of drug-target interactions at the single cell level
Source: PLoS Comput Biol. 2018 Dec 20;14(12):e1006601. doi: 10.1371/journal.pcbi.1006601 (PMC6319770; doi:10.1371/journal.pcbi.1006601)
Supplement: S1 Text — (PDF) [file pcbi.1006601.s001.pdf]

Determinants of Drug-Target Interactions at the Single Cell Level, S1 Supporting Information

Vlad Elgart[1,2]◕, Jia-Ren Lin[1,]◕, Joseph Loscalzo[1,2*],

1 Laboratory for Systems Pharmacology,

Harvard Medical School, Boston, Massachusetts, USA.

2 Department of Medicine,

Brigham and Women's Hospital, Boston, Massachusetts, USA.

◕These authors contributed equally to this work.

* jloscalzo@rics.bwh.harvard.edu, Joseph Loscalzo, M.D., Ph.D.,

Brigham and Women's Hospital, 75 Francis St., Boston, MA 02115; 617-732-6340

Almost Irreversible Dye Binding

We observed that extracellular dye removal does not affect the fluorescence signal from bound dye-DNA complexes in intact nuclei (cf., Fig. Ba and Fig. Bb). Since the major contribution of the Hoechst fluorescence corresponds to dye bound tightly to the minor groove of DNA, we concluded that this binding is virtually irreversible on the time scales of our experiments (72 hours or less).

By contrast, if we expose cells to Hoechst and then lyse them, we observe that the intensity of the fluorescence signal depends on whether extracellular dye was removed or not prior to lysis. The blue curve in Fig. C corresponds to the case where extracellular Hoechst is not removed prior to lysis. The green curve in Fig. C corresponds to the case where extracellular Hoechst is removed (and cells are resuspended in dPBS) prior to lysis. Long exposure to Hoechst prior to lysis guarantees the achievement of the steady-state levels of bound DNA in both cases prior to lysis. After lysis, the fluorescence signal intensity of bound dye drops instantaneously if extracellular Hoechst was removed prior to lysis (cf. blue and green curves). We believe this decrease corresponds to a new steady-state due to equilibration between bound DNA of lysed cells and fresh (dye-free) buffer used to re-suspend the cells.

The fact that a new equilibrium can be achieved means that binding is reversible (and that the time scale is faster than our detection limit of 1 minute) if cells are lysed. To ensure that the decrease in signal intensity is not due to a technical issue of losing cell lysate during centrifugation and re-suspension, we repeated these steps twice in a series with additional TritonX treatment, as well. The corresponding signal is depicted by the red curve in Fig. C.

Note that the time-dependence of the signals in Figs. B and C is most likely due to photobleaching and not chemical re-equilibration on the time scales of our experiments. This conclusion is supported by the similarity in time-dependent reduction of the signal in all cases,

including those involving calf thymus DNA control experiments.

Moments of Inertia, Theoretical Considerations

All of our theoretical and numerical calculations are based on an idealized model of the cell; namely, we assume radial symmetry in both shape and target distribution of the cell. Within our experimental setting, we measure fluorescence intensity emitted from a thick slice of each nucleus in the z-direction. To emulate the experimental data, we considered parameters of the idealized model that can be expressed in terms of a z-projection of physical variables. We consider the total intrinsic amount of bound DNA in the cell, ”mass” of the nucleus M_0 (that emulates I_{tot} in the experimental signal), and moment of ”inertia” M_2 with respect to the z-axis:

$$M_n = \int_V (r_x^2 + r_y^2)^{n/2} \, \rho(\vec{r}) \, \mathrm{d}\vec{r}, \tag{1}$$

where V and $\rho(\vec{r})$ are volume and target density of DNA in the cell, respectively.

For a general non-uniform but radially symmetric ”mass” distribution of the target density $\rho(r)$, we derive the following moments of inertia:

$$M_0 = \int_0^R (4\pi \, r^2) \, \rho(r) \, \mathrm{d}r, \tag{2a}$$

$$M_2 = \int_0^R \frac{2}{3} r^2 \, (4\pi \, r^2) \, \rho(r) \, \mathrm{d}r. \tag{2b}$$

The quadratures Eq. (2) are obtained by partitioning the entire sphere into thin shells of radius r where local density is uniform. The mass of each thin shell is $\Delta m = (4\pi \, r^2) \, \rho(r) \, \mathrm{d}r$ and its radius is r by construction. The first two moments of inertia of a thin spherical shell are well known, namely Δm and $\frac{2}{3} r^2 \, \Delta m$ for a thin shell of mass Δm (cf., (*2*)). Since the total moment of inertia of any object is the sum of its partitions’ moments, we can derive Eq. (2).

For time- and position-dependent concentration of free DNA $\rho = v(r,t)$ in our numerical simulation, we used Eq. (2) in order to calculate equivalents of experimentally measured $I_{tot}(t)$ and $M_2(t)$. We assume that the measured fluorescence signal is linearly proportional to the mass of bound DNA molecules within the measurement volume.

In order to compare reaction front propagation dynamics between individual nuclei, we defined the dimensionless quantity

$$\mu(t) = \frac{1}{\langle r_x^2 + r_y^2 \rangle} \frac{M_2(t)}{M_0(t)}\,, \tag{3}$$

where $\langle r_x^2 + r_y^2 \rangle$ describes the average squared radius of the *entire* nucleus along the $x - y$ plane. The usefulness of the variable μ is clear if one considers a limiting case such as a reaction-limited front propagation. In this case, diffusion is fast and all moments of inertia, as defined in Eq. (1) have the same time dependence since free dye distribution is uniform everywhere in the nucleus, and, therefore, $\rho(r,t) = \rho(t)$. This means that $\mu(t) = const$, (see Eq. (3)), which only depends on the geometry of the nucleus. Hence, we interpret the moment in time when $\mu(t)$ reaches a constant value as the terminal state of front propagation. After this time, dye incorporation occurs uniformly throughout entire nucleus. Since our definition of the dimensionless variable $\mu(t)$ includes normalization to geometric size, we can compare the dynamics of $\mu(t)$ between individual nuclei regardless of their size.

Below, we consider a few limiting cases of the result Eq. (2), which were used as references in figures throughout the article. The moment of inertia of a solid uniform shell with outer radius $R_>$ and inner radius $R_<$ is given by

$$M_2^{sh} = \frac{2}{5} \frac{R_>^5 - R_<^5}{R_>^3 - R_<^3}\, M_0. \tag{4}$$

The moment of inertia of a solid uniform disk of radius $R_>$ is given by

$$M_2^d = \langle r_x^2 + r_y^2 \rangle\, M_0 = \frac{1}{2} R_>^2\, M_0. \tag{5}$$

Hence, the thickness of the shell can be deduced from the dimensionless geometric ratio:

$$\mu^{sh} = \frac{M_2^{sh}}{\langle r_x^2 + r_y^2 \rangle M_0} = \frac{M_2^{sh}}{M_2^d} = \frac{4}{5} \frac{R_>^5 - R_<^5}{R_>^2 (R_>^3 - R_<^3)}. \tag{6}$$

For solid ball, thin shell, and thick shell, one derives, respectively

$$\mu = \begin{cases} \frac{4}{5}, & \text{if } R_< = 0 \\ \frac{4}{3}, & \text{if } (R_> - R_<) \ll R_> \\ \frac{31}{35}, & \text{if } R_> = 2R_< \end{cases} \tag{7}$$

where the condition on the right specifies each limiting case.

Comparison of Sub-Regional to Total Fluorescence Intensity

For an arbitrary sized sub-region of an ideal spherical nucleus, one can define the time-dependent sub-total fluorescence intensity $J(r,t)$. Using this notation, the total nuclear fluorescence intensity is $J(R,t) = I_{tot}(t)$, where R is the size of the nucleus. For a uniformly distributed fluorescence density $\rho(t)$ and small sub-region size $r/R \ll 1$, one derives:

$$J(r,t) \approx \rho(t) \cdot (2R) \cdot \pi r^2 \tag{8a}$$

$$J(R,t) = \rho(t) \cdot (4/3)\, \pi R^3 \tag{8b}$$

Hence, once dye is spread homogeneously throughout the nucleus, one expects a parametric plot $J(r,t)$ vs. $J(R,t)$ to have a slope of $(3/2) \cdot r^2/R^2$. By contrast, at the very initial phase of dye incorporation, one expects only a thin layer of the nucleus (close to the surface) of size $h(t)$ to be fluorescent. In this case, we derive:

$$J(r,t) \approx \rho(t) \cdot 2h(t) \cdot \pi r^2 \tag{9a}$$

$$J(R,t) = \rho(t) \cdot h(t) \cdot 4\, \pi R^2 \tag{9b}$$

Therefore, in this regime, the expected slope of the parametric curve is $(1/2) \cdot r^2/R^2$.

In this particular experiment, the dye concentration in solution was high enough ($[\mathrm{dye}] = 2\,\mu M$) to ensure that the intensity of nuclear fluorescence saturates at the steady-state. As a result, a parametric plot of the data (Fig. E) exhibits point "condensation" toward the ends of the parameterized curves, consistent with theoretical expectations. Straight line segments of the curves depicted in Figure E correspond to slopes in the limiting cases defined by Eqs. (8) and (9).

Comparison of sub-regional to total fluorescence intensity (Fig. E) demonstrates variability in the dynamics of reaction front propagation among cells. However, quantification of the dynamics based on this representation relies heavily on a uniform distribution of target density and symmetry of the nuclei. We used the pixel intensity coefficient of variation CV_p as a proxy for homogenization of dye in each individual nucleus. Indeed, once dye molecules are distributed uniformly throughout the nucleus, one expects binding kinetics to be uniform, as well [cf. Eq. (5)]. This approach can be used to infer the dynamics of dye propagation in the nucleus; however, it is fairly sensitive to the background subtraction method used.

Numerical Simulation of Reaction–Diffusion System

We used Wolfram Mathematica 11 to run simulations of nonlinear reaction–diffusion models. All models assume stationary targets (specific and non-specific) and diffusing dye molecules. All initial and boundary conditions as well as the distribution of targets inside the nucleus were assumed to be spherically symmetric. Hence, the Laplacian that describes the diffusion operator in the main text, Eq. (10), is given as

$$\nabla_x^2 u = \frac{1}{r^2}\frac{\partial}{\partial r}\left(r^2\frac{\partial u}{\partial r}\right) \tag{10}$$

in spherical coordinates. We assumed a Neumann boundary condition in the center of nucleus $r = 0$ and a Robin boundary condition imposed at the nuclear membrane $r = 1$, where 1 is a

dimensionless radius of the nucleus. Our initial condition assumed a dye-free nucleus at time $t = 0$.

For a binding model with only specific binding interactions, these initial and boundary conditions are, then,

$$u(r, 0) = 0, \quad \text{if } r < 1 \tag{11a}$$

$$v(r, 0) = c, \quad \text{if } r < 1 \tag{11b}$$

$$u^{1,0}(0, t) = 0 \tag{11c}$$

$$u^{\{1,0\}}(1, t) = Bi\,[u_{ext} - u(1, t)] \tag{11d}$$

where Bi is a corresponding Biot number (effective mass transfer coefficient). Similar boundary conditions were imposed for numerical simulation of the model describing multiple types of targets, both specific and non-specific.

Numerical Simulation of Dye Incorporation Kinetics

In what follows, we assumed that a solution to reaction-diffusion equation(s) should always exist and be unique due to the physical nature of the problem at hand. We relied on the convergence of the numerical simulation as a guide for an acceptable parameter range.

A typical MFC10A cell nucleus has an ellipsoidal geometry of the characteristic size $R_n \approx 20\,\mu m$. If we measure the length in units of $20\,\mu m$ and the dye concentration in units of μM (the characteristic concentration of dye in our setting), the resulting transformation corresponds to the introduction of dimensionless length and concentration terms into Eqs. (9)–(12). In these (dimensionless) units, the size of a nucleus is $R_n \sim 1$ and the diffusion coefficient of free dye in water is:

$$D \sim \frac{400\,\mu m^2\, sec^{-1}}{(20\,\mu m)^2} = 1\, sec^{-1} \tag{12}$$

The association rate k_{on} in these units is, then, approximately $10^2\, sec^{-1}$. The overall DNA concentration in the nucleus (estimated as the number of base pairs per total volume, [bp]) is:

$$[\text{bp}] \sim \frac{3 \cdot 10^9}{(4/3)\pi(20\,\mu m)^3} = 9 \cdot 10^{19}\, l^{-1} = 150\,\mu M \tag{13}$$

or [bp] $= 150$ in dimensionless units.

Dye binding affinity and subsequent fluorescence depend upon the DNA base pair sequence. Dye binding to an $AATT$ site is of significantly greater affinity than to other sites and results in a greater fluorescence intensity (*1*). Other investigators have estimated that such complexes in calf thymus DNA (CT) occur with a frequency of about 1 binding site per 100 base pairs (*3*). Taking this observation into account, we estimate the concentration of the available specific binding sites to be:

$$v_{max} \sim 1.5 \tag{14}$$

using the dimensionless units introduced above and in Eq. (13).

Using dimensionless units of length and concentration, one derives from Eqs. (9)–(12):

$$\mathcal{R}(u,v) = 10^2\, u\,(1.5 - v) - 10^{-1}\, v \tag{15a}$$

$$\partial_t u(r,t) = \frac{1}{r}\partial_r^2(r\,u) - \mathcal{R} \tag{15b}$$

$$\partial_t v(r,t) = \mathcal{R}(u,v) \tag{15c}$$

$$\partial_r u(r,t) = Bi\,[u_{ext} - u(r,t)]\,, \quad r = 1 \tag{15d}$$

$$u(r,0) = 0, \quad v(r,0) = 0, \quad r \leq 1 \tag{15e}$$

Here, we assume that the nucleus is a sphere of radius $R_n = 1$, and that Bi is the Biot number given by:

$$Bi = \frac{h_m R_n}{D} \tag{16}$$

The only unknown parameter in Eq. (15) is the Biot number, which can be determined by fitting the experimental data.

Upon further consideration, one realizes that the model described by Eq. (15) is inadequate. The main problem here is not only the dynamics (see below), but also the steady-state prediction based on Eq. (15). In the steady-state, the free extracellular dye concentration will be the same as the intracellular concentration, $u_{ext} = u_{st}$. Hence, the bound dye concentration in the steady-state is given by the solution:

$$\mathcal{R}(u, v) = 10^2 \, u_{st} \, (1.5 - v_{st}) - 10^{-1} \, v_{st} = 0 \tag{17}$$

$$v_{st} = 1.5 \frac{10^2 \, u_{ext}}{10^2 \, u_{ext} + 10^{-1}} \; \mu M \tag{18}$$

and is completely insensitive to u_{ext} in the range of concentrations higher than $0.01 \, \mu M$; however, we observed a great sensitivity to dye concentration in cell culture in the range of $0.1 \, \mu M - 10 \, \mu M$.

Not surprisingly, the numerical solution of Eq. (15) for a broad range of $Bi \in \{10^{-6}, 1\}$ does not produce the dynamical behavior observed in our experiments. The results of the numerical simulation for [dye] = $1 \, \mu M$ are depicted in Figures Ka and Kb. Note first that the time dependence of the experimental traces does not approximate linear behavior. Second and more importantly, we observed a high degree of correlation between the relaxation rates for I_{tot} and M_2, which is not the case for the numerical simulation of Eq. (15).

The incorporation of continuous dye depletion into our model, $u_{ext} = u_{ext}(t)$, does not remedy this situation (Figs. Kc and Kd). In the case of rapid dye depletion, the linear behavior in Fig. Ka appears more realistic (Fig. Kc); however, the kinetics of the moment of inertia is still largely independent of the Biot number in this setting. In addition, the dye depletion rate cannot be faster than the typical rate of uptake of dye by an individual cell. Hence, for a significant population of cells, we can expect the behavior depicted in Figures Ka and Kb. Thus, given

these findings, the model described by Eq. (15) is an inadequate fit to the experimental data. Despite this shortcoming, it is very instructive to investigate the dynamic behavior prescribed by the model of Eq. (15) in order to refine the model further and best fit the experimental data.

Numerical Estimate of the Concentration of Non-Specific Binders in the Cell

In order to estimate the amount of non-specific species interacting with Hoechst dye, we use steady-state experimental data depicted in Figures Ga and Gb. We consider a simple reaction scheme, which is essentially a homogeneous version of Eqs. (34)–(38) since we are interested only in steady-state conditions in which all targets are uniformly distributed within the cell:

$$u\,(c_i - v_i) = K_d^i\, v_i \tag{19a}$$

$$u + \sum_{i=1}^{2} v_i = dye \tag{19b}$$

$$s = \sum_{i=1}^{2} a_i\, v_i \tag{19c}$$

Here u and dye are the concentrations of free and total dye molecules in solution, respectively; and v_i and c_i are concentrations of bound and total species of class i (i.e., specific and non-specific binders, respectively). The K_d^i is the dissociation constant and a_i is fluorescence intensity for each species. Finally, s is a "signal," or the resulting fluorescence we observe experimentally. We assume here that all binding reactions are binary and that there are only two different classes of species for simplicity.

Several variables in system Eq. (19) can be measured directly, namely, signal s, dye concentrations u, and dye. Ideally, we have to fit four parameters that presumably remain constant under all experimental conditions, namely, the dissociation constants K_d^i and total species concentrations c_i. Since the system Eq. (19) is underdetermined for a given condition (for 2 species

there are 4 equations and 8 unknowns), the dye/cell titration data can be used to estimate the binding parameters. In Figure P, we compare the experimental data with the fitted simulation of Eq. (19). This estimate supports the assumptions used for the numerical reaction–diffusion simulations in the previous section, at least within an order of magnitude.

Mean-Field Solution to Autonomous Binary Reaction Model

An implicit solution of a differential equation of the following form:

$$\frac{d}{dt} v = -k \cdot (v - \lambda_1)(v - \lambda_2), \tag{20a}$$

$$v(0) = c, \tag{20b}$$

is given by:

$$t = \int_c^{v(t)} \mathrm{d}z \; [k \cdot (v - \lambda_1)(v - \lambda_2)]^{-1} = \frac{1}{k \cdot (\lambda_2 - \lambda_1)} \log \left[\frac{z - \lambda_2}{z - \lambda_1} \right]_{z=c}^{z=v(t)} . \tag{21}$$

Hence, a characteristic relaxation rate of dye binding dynamics is given by:

$$\beta = k \cdot |\lambda_2 - \lambda_1|. \tag{22}$$

In what follows, we demonstrate that for autonomous mean-field dye-DNA binding models involving any number of interacting species, the problem can be reduced to Eq. (20) and, hence, have a solution given by Eq. (21).

Indeed, for a single species of dye binding molecules, the reaction scheme is simply:

$$U + V \underset{k_{\mathrm{off}}}{\overset{k_{\mathrm{on}}}{\rightleftharpoons}} UV. \tag{23}$$

Where V represent *free* DNA that can bind to dye molecule U.

The reaction dynamics is described by:

$$\frac{d}{dt}\, v(t) = -k_{\rm on}\, u(t) \cdot v(t) + k_{\rm off}\, [c - v(t)], \tag{24a}$$

$$u(t) - v(t) = const = u_0 - c, \tag{24b}$$

$$v(0) = c, \tag{24c}$$

where u and v are concentrations of species U and V, respectively.

It is straightforward to map Eq. (24a) onto Eq. (20) by identifying:

$$a_{1,2} = -\frac{1}{2}(K_d + u_0 - c) \pm \sqrt{(K_d + u_0 - c)^2 + 4\, K_d\, c}, \tag{25}$$

and $k = k_{\rm on}$. By construction, λ_1 corresponds to the steady-state solution of Eq. (24a).

Hence, the characteristic relaxation rate of reaction dynamics in the case of a single binding species is given by:

$$\beta = k \cdot |\lambda_2 - \lambda_1| = k_{\rm on} \cdot \sqrt{(K_d + u_0 - c)^2 + 4\, K_d\, c}. \tag{26}$$

The explicit time dependence of free DNA concentration, $v(t)$, can be derived by resolving the implicit solution, Eq. (21), as:

$$\left[\frac{v(t) - \lambda_2}{v(t) - \lambda_1}\right] = \left[\frac{c - \lambda_2}{c - \lambda_1}\right] \exp(\beta\, t)\,. \tag{27}$$

Using Eq. (27) one can resolve $v(t)$ explicitly.

We note here that the derived expression for the relaxation rate Eq. (26) would, of course, also describe the relaxation rate of dye uptake (due to conservation of total dye molecules). Dye depletion is mostly driven by buffering by non-specific binding [cf., Eq. (30)]. Since we wish to determine the dynamics of intracellular dye concentration and not simply in the nucleus,

concentrations are calculated per well volume $V \sim 150\,\mu l$:

$$c_n \sim (N_{cells}\,N_{bp})/(VN_A) \sim 1\,\mu M \tag{28}$$

where $N_{cells} \sim 3 \cdot 10^4$ is a typical number of cells per well (96 well plate), and $N_{bp} \sim 3 \cdot 10^9$ and N_A are the number of base pairs in the genome and Avogadro's number, respectively. Combining Eqs. (26) and (28), it is possible to estimate the dye uptake rate based on a mean-field model.

Let us now turn to a system that involves multiple interacting species. In this case, one can describe the interactions as follows:

$$U + V_i \underset{k^i_{\text{off}}}{\overset{k^i_{\text{on}}}{\rightleftharpoons}} UV_i. \tag{29}$$

Here V_i represent different stationary species i that can bind to a diffusing molecule (walker) U and immobilize it. We restrict our analysis to two species of stationary molecules, specific $i = s$ and non-specific $i = n$. We will assume that concentrations of non-specific species, $v_i = [V_i]$, and reaction rates, k^i_{off}, k^i_{on}, satisfy the following relationships:

$$v_s \ll v_n, \tag{30a}$$

$$k^s_{\text{off}} \ll k^n_{\text{off}}. \tag{30b}$$

$$k^s_{\text{on}} = k^n_{\text{on}} = k_{\text{on}}. \tag{30c}$$

The first two assumptions in Equations (30a) and (30b) can be reasoned rather straightforwardly based on the operational definition of specificity. The last assumption in Equation (30c) reflects the fact that the binding rate is driven by the diffusion rate and molecular weight of the same species U and, hence, is similar for all binding reactions.

The dynamics of the reaction model Equation (29) is described by

$$\frac{d}{dt}\, v_i(t) = -k^i_{\mathrm{on}}\, u(t) \cdot v_i(t) + k^i_{\mathrm{off}}\, [c_i - v_i(t)], \quad i = n, s \tag{31a}$$

$$u(t) - \sum_{i=n,s} v_i(t) = const = u_0 - \sum_{i=n,s} c_i, \tag{31b}$$

$$v_i(0) = c_i \quad i = n, s, \tag{31c}$$

where u is the concentration of a given mobile species $u = [U]$.

Numerical simulations of Eq. (31) suggest that for abundant non-specific species, $c_n > u_0$, there are two characteristic regimes of Eq. (31) dynamics. These behaviour of these two regimes is shown in Fig. Ja (blue curve) and can be interpreted as follows: On very short time scales (first regime), free dye quickly becomes bound by interacting species V_i. The second regime corresponds to much slower re-distribution of bound dye molecules between species V_i. In the first regime, the dynamics is driven by binding only (dissociation rate is negligible in this regime). Hence for equal association rates, Eq. (30c), one expects that free species V_i concentrations $v_i(t)$ are proportional to each other in this regime. Indeed, under these assumptions, we can derive from Eq. (31):

$$\frac{d}{dt}\, v_i(t) \approx -k_{\mathrm{on}}\, u(t) \cdot v_i(t), \quad i = n, s \tag{32a}$$

$$\frac{dv_i}{v_i} \approx -k_{\mathrm{on}}\, u(t), \quad i = n, s. \tag{32b}$$

Hence, we derive for this regime a relationship:

$$\frac{v_n(t)}{v_s(t)} \approx const = \frac{c_n}{c_s}, \tag{33}$$

which is, of course, generalizable to greater amounts of interacting species.

In the second regime, dissociation becomes important. However, if the amount of remaining free dye is small, we can derive a second conservation law of the total amount of bound dye

molecules that relates concentrations $v_i(t)$ in linear fashion. The simplest way to do so is to substitute an ansatz into Equation (31).

$$v_n(t) = g_1 + g_2\, v_s(t), \tag{34}$$

The coefficients g_1 and g_2 can be resolved by demanding consistency between the resulting equations in Eq. (31):

$$g_1\, k_{\rm off}^{n} + g_2\, c_s k_{\rm off}^{s} + g_1\,[g_1 + u_0 - c_n - c_s]\, k_{\rm on} = c_n\, k_{\rm off}^{n} \tag{35a}$$

$$g_1\, k_{\rm on} + g_2\,(k_{\rm off}^{n} - k_{\rm off}^{s} + g_1\, k_{\rm on}) = 0. \tag{35b}$$

The algebraic equations Eq. (35) have three possible solutions, with one being physically meaningful.

To demonstrate that the conservation laws for both regimes are correct, we considered a parametric plot of concentrations $\{v_s, v_n\}$ determined by numerical simulation of Eq. (31). The result, shown in Fig. Jb, illustrates the linear dependence of $v_s(t)$ and $v_n(t)$ in the two regimes (blue curve). The linear solutions of conservation laws Eqs. (33) and (34) (dashed black and red lines, repectively) fit these two regimes very well. The intersection of two linear curves Eqs. (33) and (34) allows one to estimate the transition point between two regimes:

$$v_s(\tau) = \frac{g_1}{c_n/c_s - g_2}, \tag{36}$$

where τ is a transition time point between two regimes.

Note that owing to the conservation law Eq. (34), a full dynamical model Eq. (31) can be reduced to an effective single variable (e.g., v_s) differential equation in both regimes. In both cases, the resulting equation is precisely the same type as for an equation involving a single interacting species Eq. (20) considered above. The only difference is the renormalization in

binding/dissociation rates and the initial conditions. In particular, the initial conditions for the slow second regime relaxation is given by Eq. (36). Hence, one can derive the explicit temporal dependence of $v_i(t)$ in these regimes. The resulting analytical approximation for the second (slow) regime is shown in Fig. Ja (dashed red curve).

Finally, we derive renormalized reaction rates, initial condition, and relaxation rate β for specific binding due to the existence of non-specific interactions, with the following relationships for renormalized quantities (marked with asterisks):

$$k_{\text{on}}^* = k_{\text{on}}(1 + g_2)\,, \tag{37a}$$

$$k_{\text{off}}^* = k_{\text{off}}\,, \tag{37b}$$

$$c_s^* = c_s\,, \tag{37c}$$

$$u_0^* = \frac{g_1 - c_n + g_2 c_s + u_0}{1 + g_2}\,, \tag{37d}$$

$$\beta = k_{\text{on}}\sqrt{((g_1 - c_n - c_s + u_0) + K_d^s)^2 + 4(g_2 + 1)c_s K_d^s}\,. \tag{37e}$$

Under the assumptions of Eq. (30), the g_2 coefficient is negative and has a value close to -1. Hence, the effective binding rate k_{on}^* is significantly smaller compared to the microscopic binding rate k_{on} by roughly a factor c_s/c_n. The change in the renormalized initial dye concentration is small and also scales as c_s/c_n. The remainder of the parameters have bare values under renormalization conditions (integrating out the degrees of freedom of the non-specific species).

Data Availability

Time resolved microscopy image datasets for all single cell experiments are publicly available using link below:

https://omero.hms.harvard.edu/webclient/?show=screen-1006

References and Notes

1. Sophia Y Breusegem, Robert M Clegg, and Frank G Loontiens. Base-sequence specificity of hoechst 33258 and DAPI binding to five (A/T)4 DNA sites with kinetic evidence for more than one high-affinity hoechst 33258-AATT complex. *J. Mol. Biol.*, 315(5):1049–1061, 1 February 2002.

2. L D Landau and E M Lifshitz. Mechanics, vol. 1. *Course of Theoretical Physics*, 3, 1976.

3. Frank G Loontiens, Peter Regenfuss, Annelies Zechel, Lieve Dumortier, and Robert M Clegg. Binding characteristics of hoechst 33258 with calf thymus DNA, poly [d (AT)] and d (CCGGAATTCCGG): multiple stoichiometries and determination of tight binding with a wide spectrum of site affinities. *Biochemistry*, 29(38):9029–9039, 1990.

List of Figures

A (a) Normalized fluorescence intensity, I/I^*, of formalin-fixed HeLa cells measured by microplate reader for Hoechst dye concentration of $8\,\mu g/ml$. Here I^* is the intensity at 3 hours of measurement, $I^* = I(180\,min)$. Cell density is $\sim 3 \cdot 10^4\,cells/well$. Extrapolated half-lives of kinetics (using spline interpolation) are $T_{1/2}$ = 14.3, 11.2, and 3.2 minutes for [Digitonin] = 0, 12.5 and $50\,\mu g/ml$, respectively. (b) Time course of fluorescence intensity of cells treated with Triton X-100 (0.1%), with the remainder of the conditions as in (a) Extrapolated half-lives of kinetics (using spline interpolation) are $T_{1/2}$ = 4.1, 4.1, and 3.5 minutes for [Digitonin] = 0, 12.5 and $50\,\mu g/ml$, respectively. . . . 20
B Decay of cell fluorescence intensity, I, in the (a) absence or (b) presence of dye (at the same concentrations) in solution. 21
C Decay of cell lysate fluorescence intensity, I, for samples initially incubated with $4\,\mu g/ml$ dye. Calf thymus DNA (CT) concentration is $12\,\mu g/ml$, cell density is $\sim 1.2 \cdot 10^6\,cells/ml$. The chase buffer was either dPBS or dPBS + 0.001% Triton X-100. The estimated loss of cell lysate due to re-suspension was $\sim 10\%$. 22
D Dye incorporation dynamics at different positions in typical spheroidal (circular) MFC10A nuclei. Dye concentration is $16\,\mu M$; magnification is 60x. 23
E Time traces of individual nuclear and sub-nuclear dye incorporation, [dye] $=$ $2\,\mu M$, plotted parametrically. The sub-nuclear region is 10% of the area of the whole nucleus, $r^2/R^2 = 0.1$. 24
F (a) Relaxation rate $\beta_{\rm Itot}$ of I_{tot} vs. relaxation rate $\beta_{\rm MI}$ of moment of inertia M_2 , for individual cells, [dye] = $16\,\mu M$. (b) Relaxation rate β of I_{tot} for different dye concentrations, population average. 25
G Fluorescence intensity I of (a) suspended cells and (b) equivalent concentration of calf thymus DNA (CT) extrapolated from titration dataset. (c) Fluorescence intensity I of supernatant incubated with $100\,\mu g/ml$ CT. Initial dye concentrations incubated with cells are listed in the legend. (d) Concentration of dye taken up by cells was determined using CT standard. 26
H Fluorescence intensity, I, of calf thymus DNA (CT) incubated with Hoechst dye at different relative concentrations. 27
I Hoechst dye fluorescence intensity vs. concentration using calf thymus DNA (CT) (concentration, $100\ \mu g/ml$). 28

J (a) Comparison of time dependence of free specific DNA species (numerical simulation, blue solid curve) and its analytic approximation (red dashed curve). Simulation parameters: $k_{\rm on} = 100, k^n_{\rm off} = 1.1, k^s_{\rm off} = 0.033, u_0 = 100, c_1 = 120, c_2 = 1.5$, where concentration dependent parameters are measured in $\mu\, M$ units and time dependent parameters are measured in sec. (b) Parametric plot of the concentrations $\{v_s(t), v_n(t)\}$, numerical simulations (blue solid curve) and analytical prediction based on conservation laws for two regimes Eqs. (33) and (34) (red dashed lines). Numerical simulation of squared displacement relative to an initial position of a tracer molecule diffusing in the 3d space: (c) no binding interactions, $k_+ = 0$ (d) $k_+ > 0$. Flat lines correspond to a period of tracer trajectory while bound to immobile species. (e) Mean squared displacement averaged over many trajectories and realizations of initial distribution of interacting species with two different k_+ values. 29
K (a) Numerical simulation (fixed external concentration u_{ext}) of overall dye incorporation per unit volume for different Biot numbers, $u_{ext} = 1\,\mu M$. (b) Moment of inertia M_2 as a function of time, same conditions as in (a). (c) Numerical simulation of overall dye incorporation per unit volume for different Biot numbers, $u_{ext} = 1\,\mu M\ \exp(-3 \cdot 10^{-4} sec^{-1}\, t)$. (d) Moment of inertia M_2 as a function of time, same conditions as in (c). 30
L Histogram of $I_{tot}(T)$, where T is the final time point of measurement. Live cells, dye concentration is $2\,\mu M$ 31
M Traces of population average intensity, I_{tot} , for different initial dye concentrations (shown in legend) at the first addition of dye in the double incubation experiment and either (a) [dye$_2$] = $0.125\,\mu M$ or (b) [dye$_2$] = $1\,\mu M$ dye concentration for the second addition. 32
N Statistics (at steady-state) of fluorescence intensity, I_{tot} : (a, b) population mean of G_1 and G_2 phases correspondingly for different dye concentrations $[dye_1]$ in the first phase of dye addition. Different curves correspond to concentrations of dye in the second phase of dye addition. (c, d) coefficient of variation of G_1 and G_2 subpopulations, same conditions as above. 33
O (a, b): Traces of normalized intensity, I^*_{tot}, [dye] = $1\,\mu M$, for G_1 and G_2 subpopulations. 34
P Experimental data (a) of observed cell fluorescence and extrapolated free dye concentration compared to simulation (b) using parameters: $K^1_d = 0.1$, $K^2_d = 2$ μg/ml, $c^1 = 1.7$, $c^1 = 34$ pg/cell. 35
Q Population average I_{tot} time dependences for different doxorubicin concentrations in fixed cell culture, (a) [dye] = $8\,\mu M$ and (b) [dye] = $0.25\,\mu M$. Population average CV_p time dependences for different doxorubicin concentrations in fixed cell culture, same conditions as in (a, b). 36

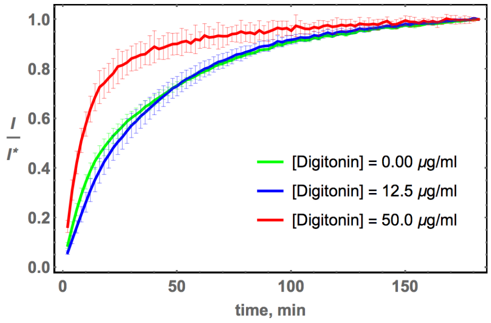

(a)

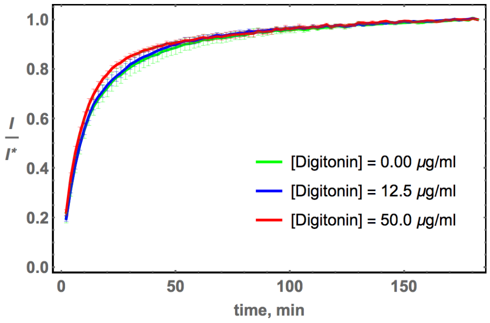

(b)

Figure A: (a) Normalized fluorescence intensity, I/I^*, of formalin-fixed HeLa cells measured by microplate reader for Hoechst dye concentration of $8\,\mu g/ml$. Here I^* is the intensity at 3 hours of measurement, $I^* = I(180\,min)$. Cell density is $\sim 3 \cdot 10^4\,cells/well$. Extrapolated half-lives of kinetics (using spline interpolation) are $T_{1/2}$ = 14.3, 11.2, and 3.2 minutes for [Digitonin] = 0, 12.5 and 50 $\mu g/ml$, respectively.
(b) Time course of fluorescence intensity of cells treated with Triton X-100 (0.1%), with the remainder of the conditions as in (a) Extrapolated half-lives of kinetics (using spline interpolation) are $T_{1/2}$ = 4.1, 4.1, and 3.5 minutes for [Digitonin] = 0, 12.5 and $50\,\mu g/ml$, respectively.

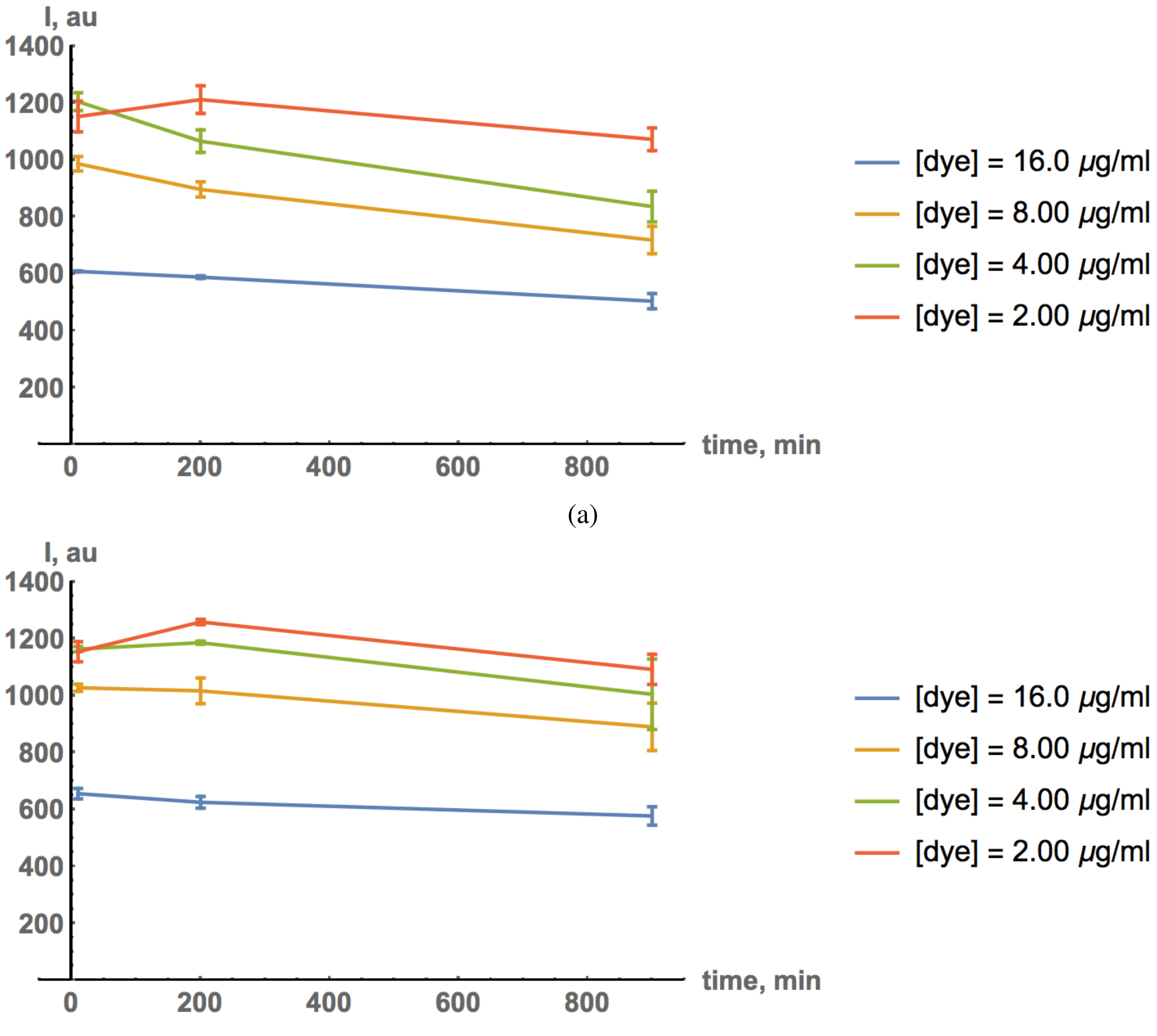

(a)

(b)

Figure B: Decay of cell fluorescence intensity, I, in the (a) absence or (b) presence of dye (at the same concentrations) in solution.

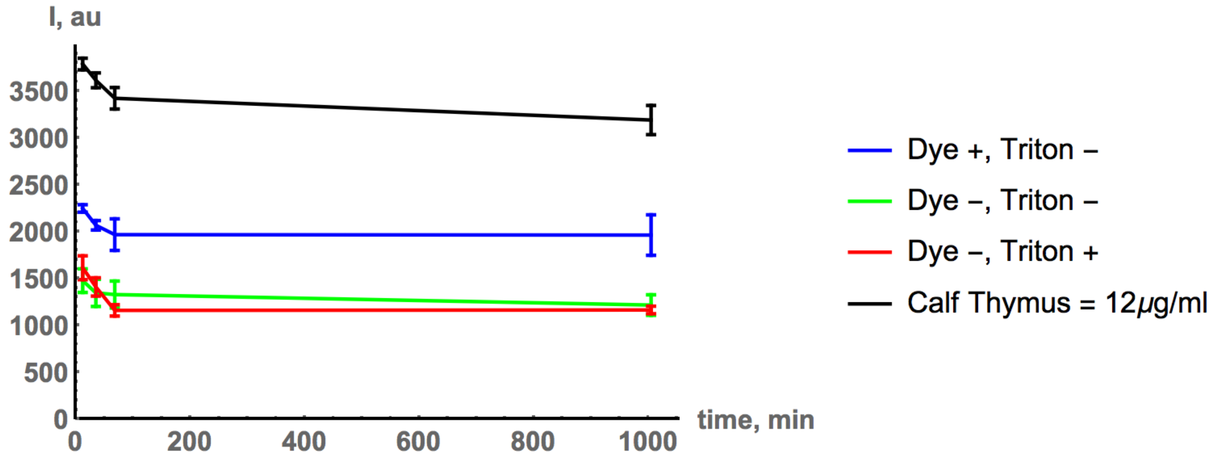

Figure C: Decay of cell lysate fluorescence intensity, I, for samples initially incubated with $4\,\mu g/ml$ dye. Calf thymus DNA (CT) concentration is $12\,\mu g/ml$, cell density is $\sim\ 1.2 \cdot 10^6\,cells/ml$. The chase buffer was either dPBS or dPBS + 0.001% Triton X-100. The estimated loss of cell lysate due to re-suspension was $\sim 10\%$.

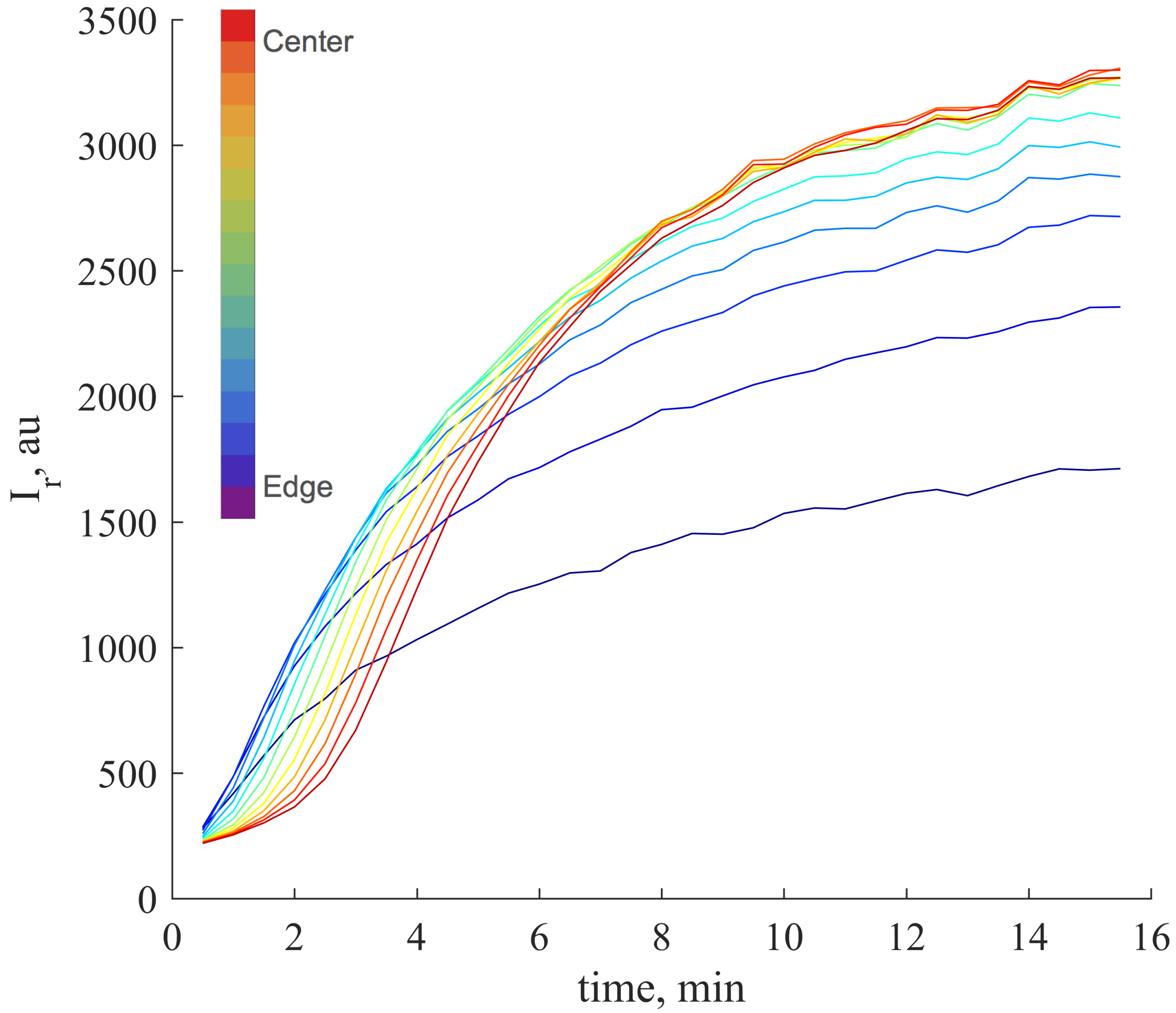

Figure D: Dye incorporation dynamics at different positions in typical spheroidal (circular) MFC10A nuclei. Dye concentration is $16\,\mu M$; magnification is 60x.

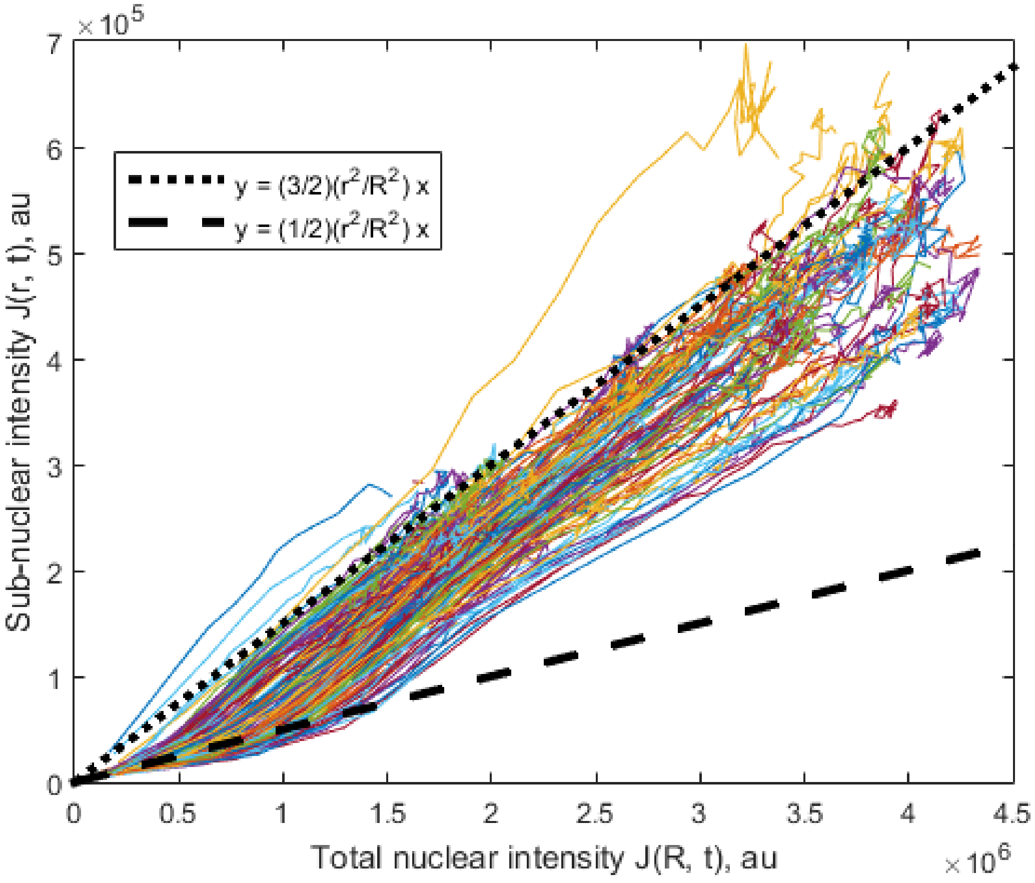

Figure E: Time traces of individual nuclear and sub-nuclear dye incorporation, [dye] $= 2\,\mu M$, plotted parametrically. The sub-nuclear region is 10% of the area of the whole nucleus, $r^2/R^2 = 0.1$.

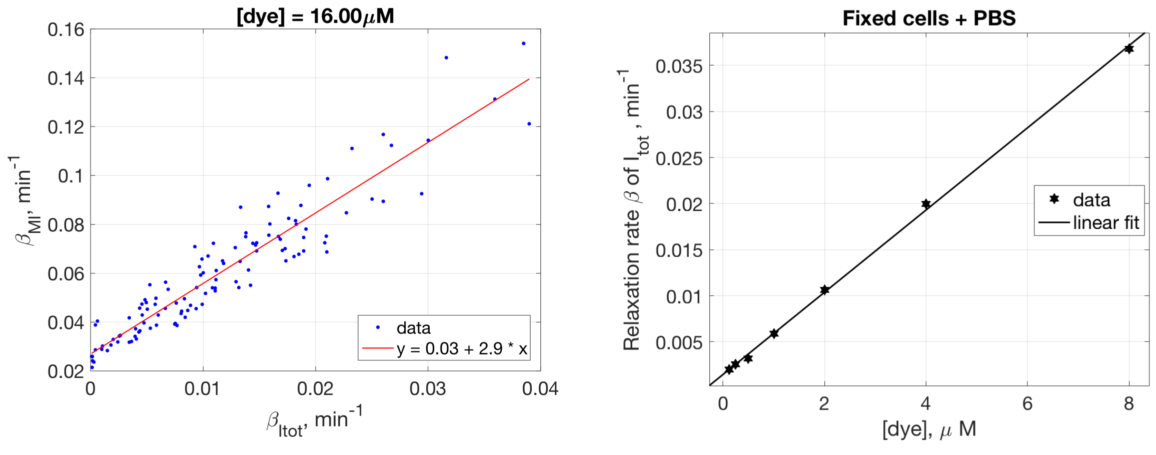

(a) (b)

Figure F: (a) Relaxation rate $\beta_{\rm Itot}$ of I_{tot} vs. relaxation rate $\beta_{\rm MI}$ of moment of inertia M_2 , for individual cells, [dye] = $16\,\mu M$.
(b) Relaxation rate β of I_{tot} for different dye concentrations, population average.

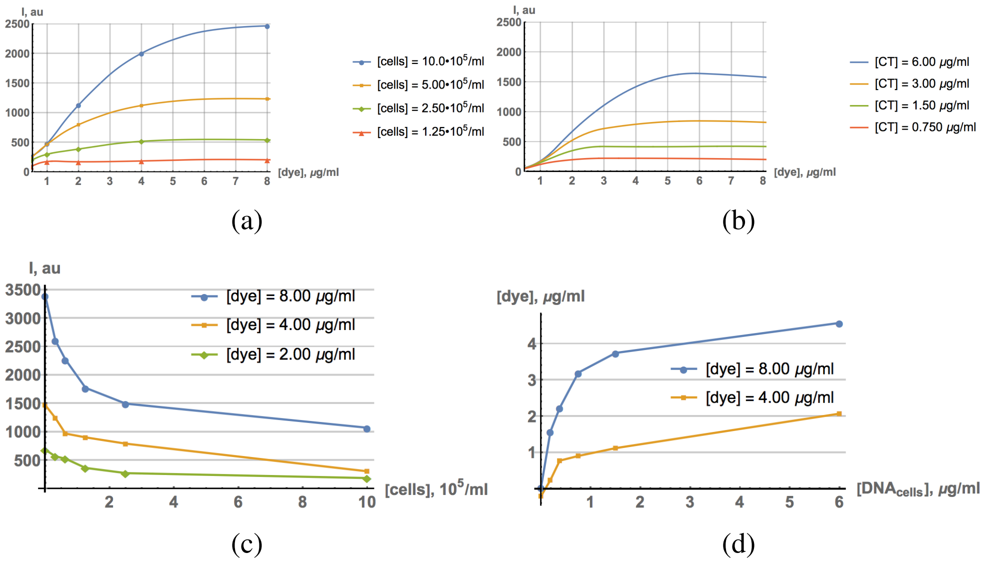

Figure G: Fluorescence intensity I of (a) suspended cells and (b) equivalent concentration of calf thymus DNA (CT) extrapolated from titration dataset.
(c) Fluorescence intensity I of supernatant incubated with $100\,\mu g/ml$ CT. Initial dye concentrations incubated with cells are listed in the legend.
(d) Concentration of dye taken up by cells was determined using CT standard.

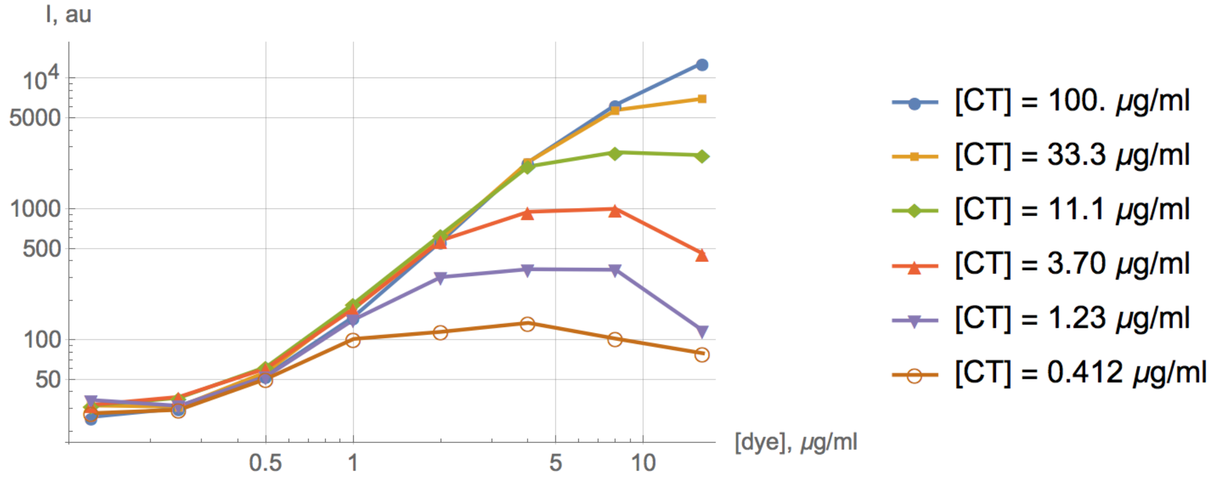

Figure H: Fluorescence intensity, I, of calf thymus DNA (CT) incubated with Hoechst dye at different relative concentrations.

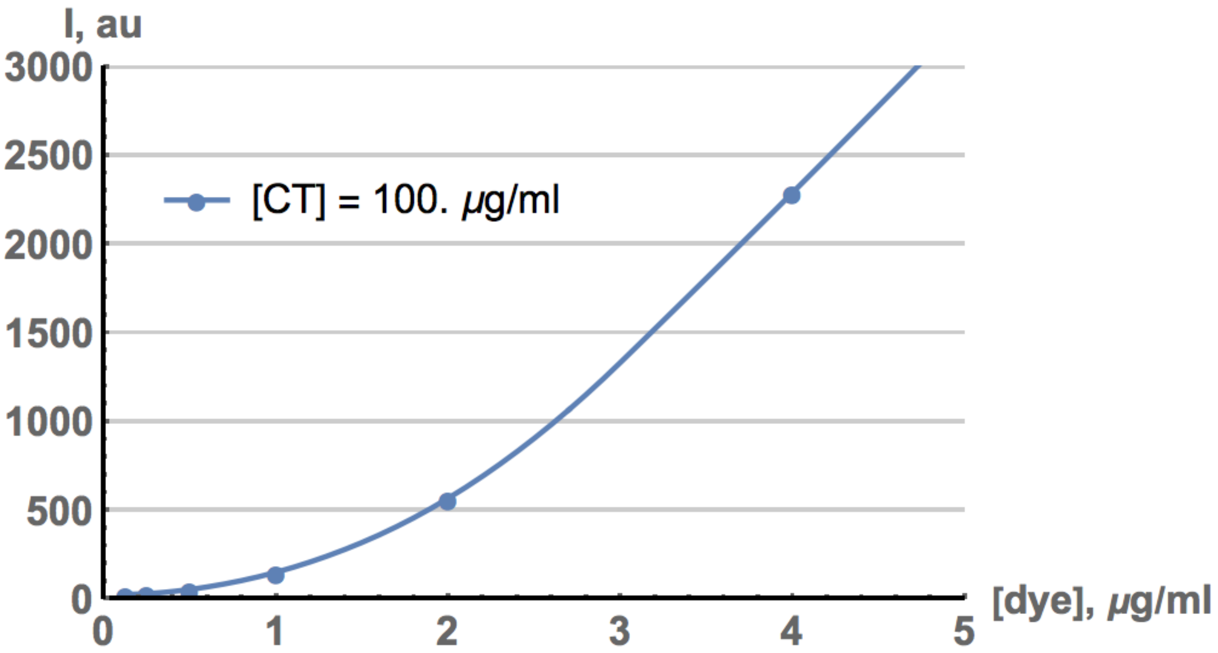

Figure I: Hoechst dye fluorescence intensity vs. concentration using calf thymus DNA (CT) (concentration, $100\ \mu g/ml$).

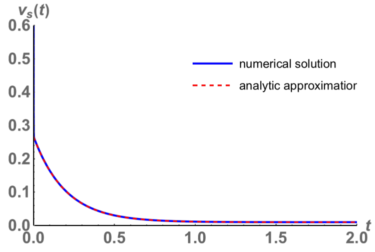

(a)

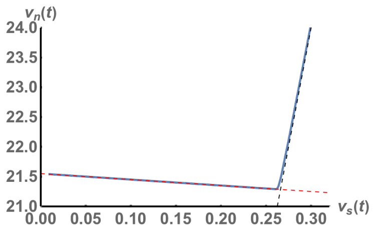

(b)

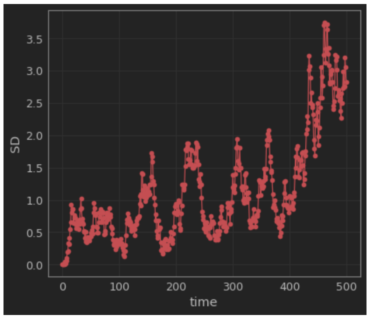

(c)

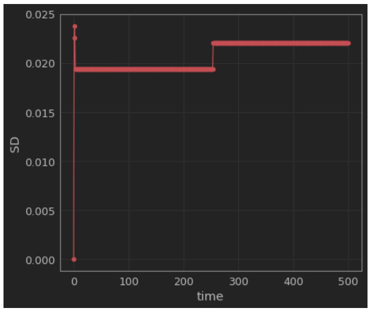

(d)

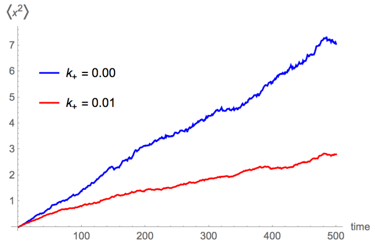

(e)

Figure J: (a) Comparison of time dependence of free specific DNA species (numerical simulation, blue solid curve) and its analytic approximation (red dashed curve). Simulation parameters: $k_{\text{on}} = 100, k^n_{\text{off}} = 1.1, k^s_{\text{off}} = 0.033, u_0 = 100, c_1 = 120, c_2 = 1.5$, where concentration dependent parameters are measured in μM units and time dependent parameters are measured in sec.
(b) Parametric plot of the concentrations $\{v_s(t), v_n(t)\}$, numerical simulations (blue solid curve) and analytical prediction based on conservation laws for two regimes Eqs. (33) and (34) (red dashed lines).
Numerical simulation of squared displacement relative to an initial position of a tracer molecule diffusing in the 3d space: (c) no binding interactions, $k_+ = 0$ (d) $k_+ > 0$. Flat lines correspond to a period of tracer trajectory while bound to immobile species.
(e) Mean squared displacement averaged over many trajectories and realizations of initial distribution of interacting species with two different k_+ values.

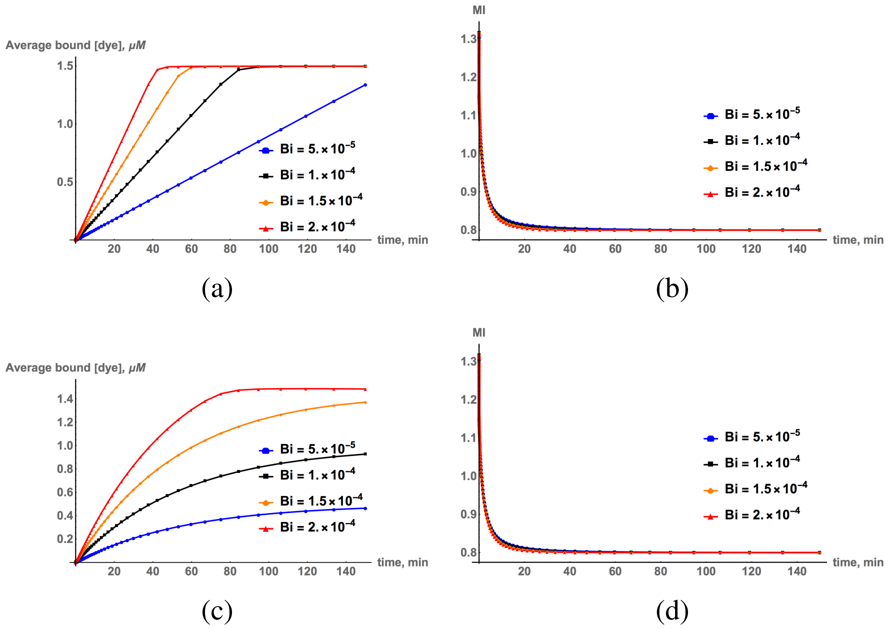

Figure K: (a) Numerical simulation (fixed external concentration u_{ext}) of overall dye incorporation per unit volume for different Biot numbers, $u_{ext} = 1\,\mu M$. (b) Moment of inertia M_2 as a function of time, same conditions as in (a).
(c) Numerical simulation of overall dye incorporation per unit volume for different Biot numbers, $u_{ext} = 1\,\mu M\ \exp(-3 \cdot 10^{-4} sec^{-1}\, t)$. (d) Moment of inertia M_2 as a function of time, same conditions as in (c).

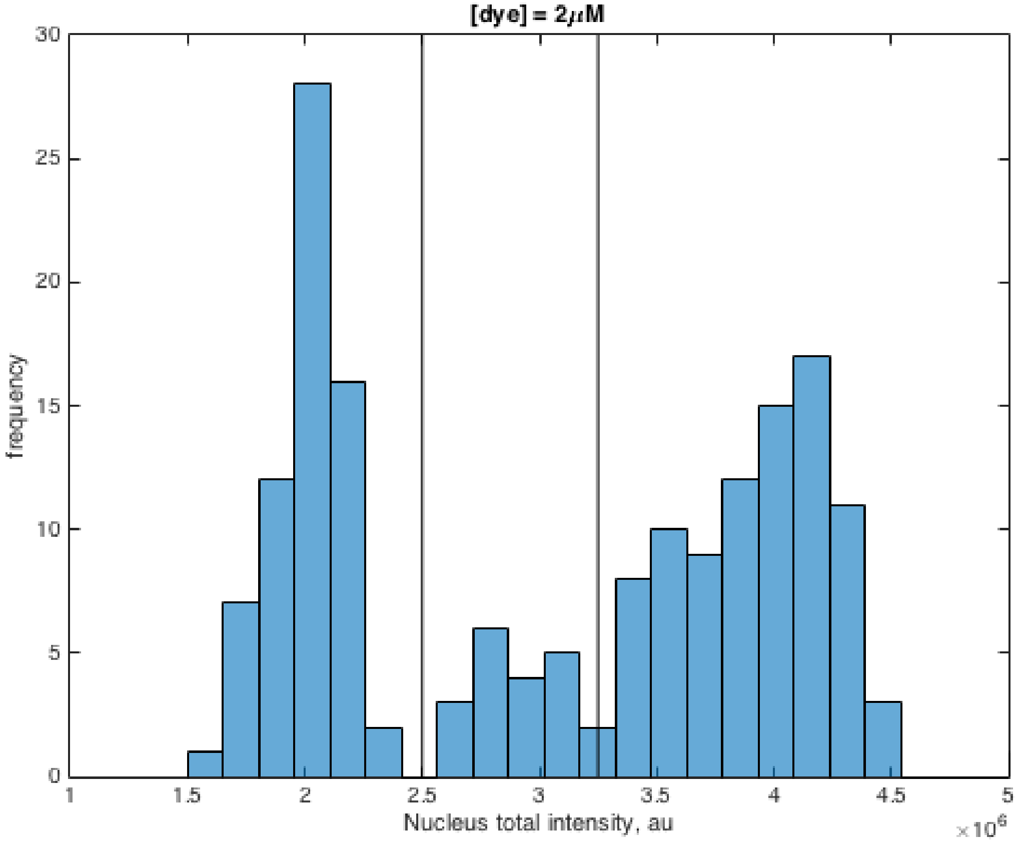

Figure L: Histogram of $I_{tot}(T)$, where T is the final time point of measurement. Live cells, dye concentration is $2\,\mu M$

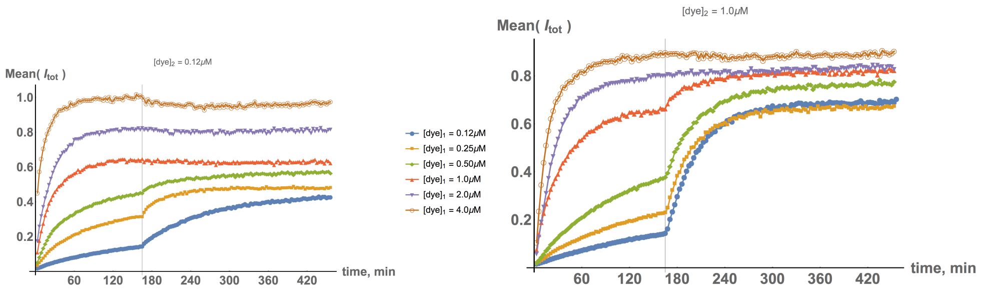

(a) (b)

Figure M: Traces of population average intensity, I_{tot} , for different initial dye concentrations (shown in legend) at the first addition of dye in the double incubation experiment and either (a) [dye$_2$] = $0.125\,\mu M$ or (b) [dye$_2$] = $1\,\mu M$ dye concentration for the second addition.

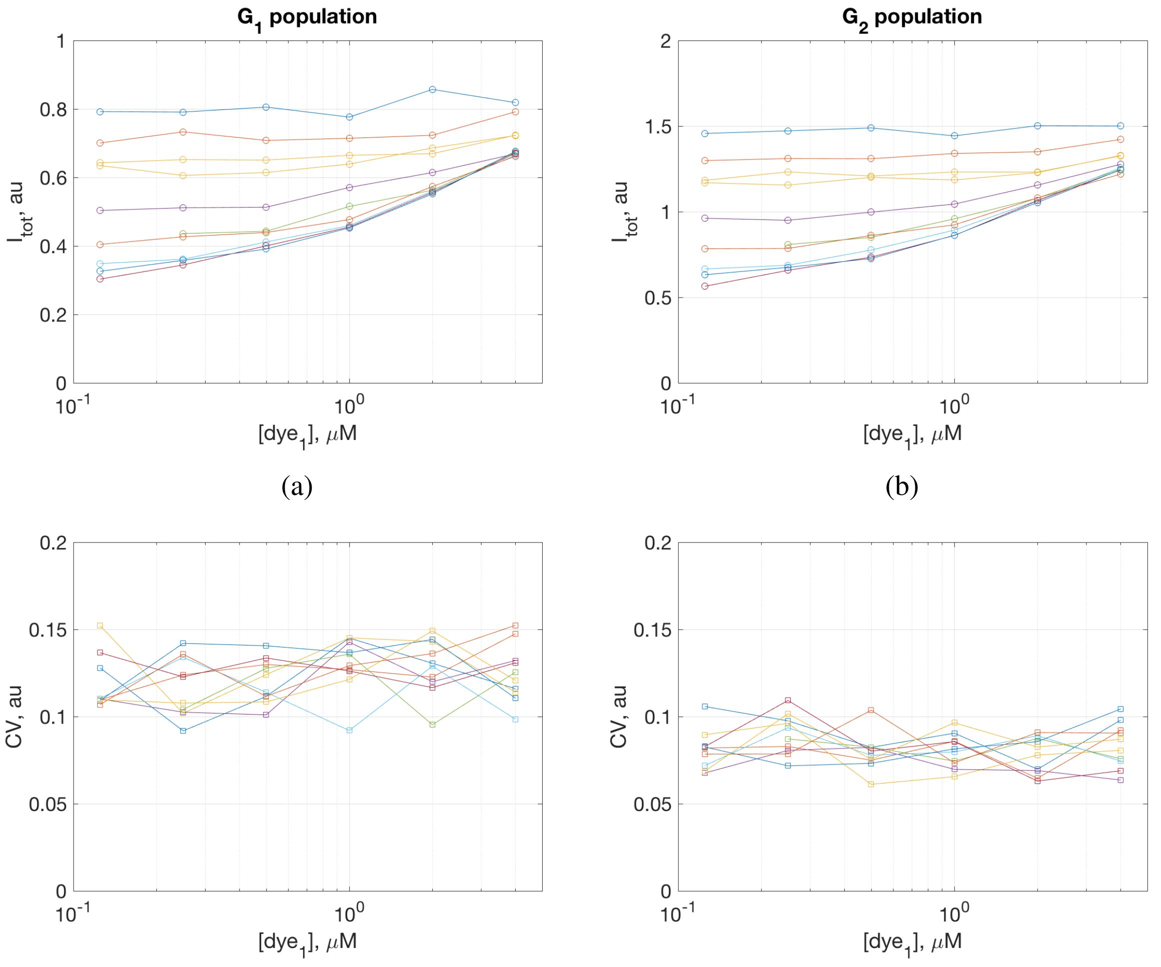

(a) (b)

(c) (d)

Figure N: Statistics (at steady-state) of fluorescence intensity, I_{tot} : (a, b) population mean of G_1 and G_2 phases correspondingly for different dye concentrations $[dye_1]$ in the first phase of dye addition. Different curves correspond to concentrations of dye in the second phase of dye addition.

(c, d) coefficient of variation of G_1 and G_2 subpopulations, same conditions as above.

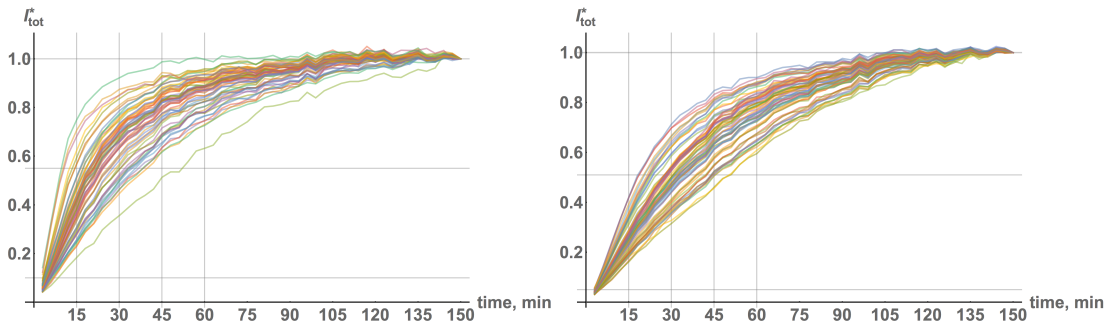

(a) (b)

Figure O: (a, b): Traces of normalized intensity, I^*_{tot}, [dye] = $1\,\mu M$, for G_1 and G_2 subpopulations.

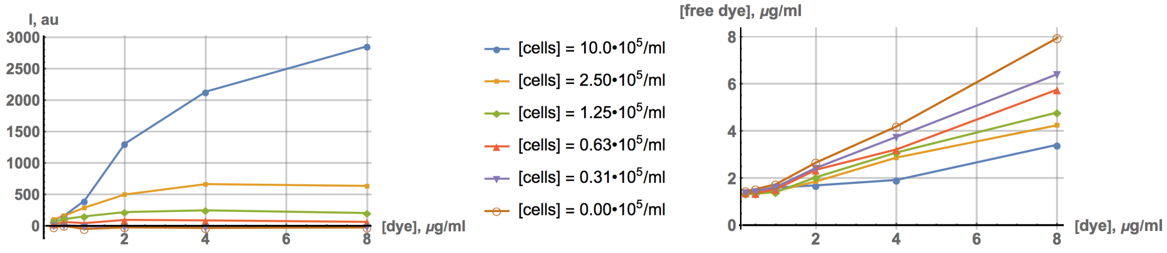

(a)

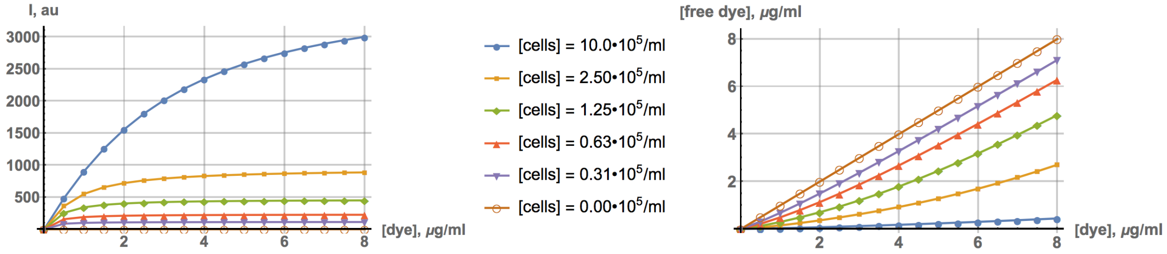

(b)

Figure P: Experimental data (a) of observed cell fluorescence and extrapolated free dye concentration compared to simulation (b) using parameters: $K_d^1 = 0.1,\ K_d^2 = 2$ μg/ml, $c^1 = 1.7,\ c^1 = 34$ pg/cell.

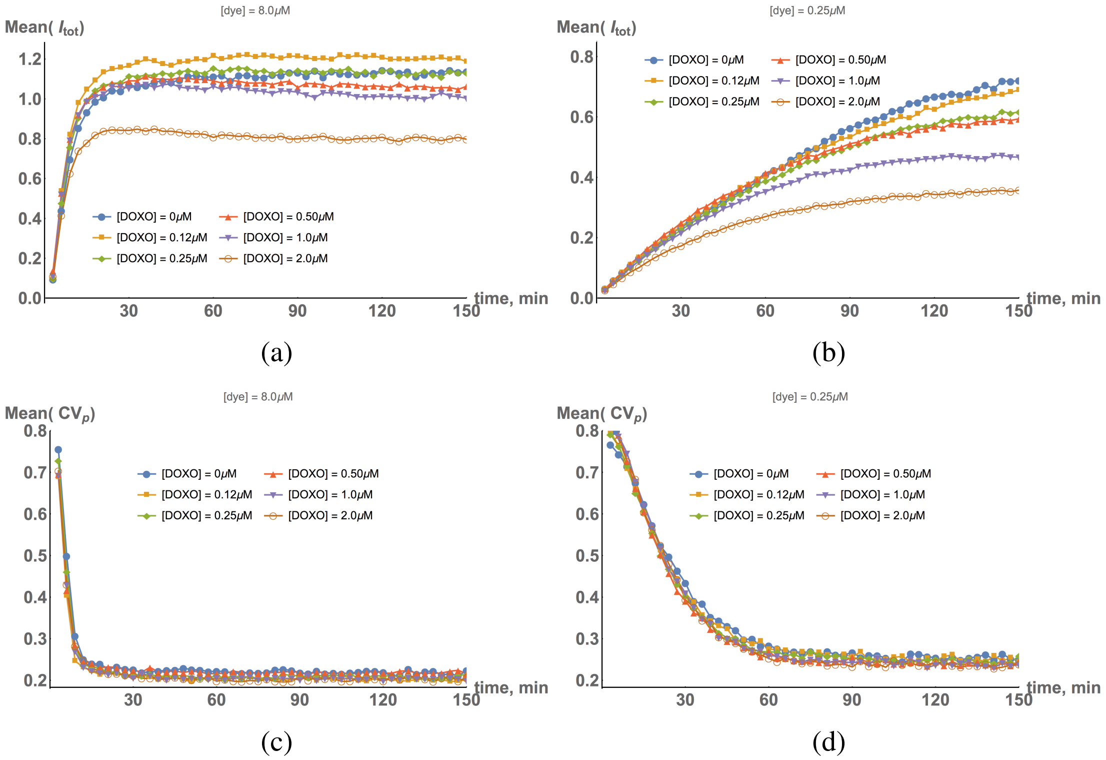

Figure Q: Population average I_{tot} time dependences for different doxorubicin concentrations in fixed cell culture, (a) [dye] = $8\,\mu M$ and (b) [dye] = $0.25\,\mu M$.
Population average CV_p time dependences for different doxorubicin concentrations in fixed cell culture, same conditions as in (a, b).
