# Supplementary material for: Determinants of drug-target interactions at the single cell level
Source: PLoS Comput Biol. 2018 Dec 20;14(12):e1006601. doi: 10.1371/journal.pcbi.1006601 (PMC6319770; doi:10.1371/journal.pcbi.1006601)
Supplement: S1 File — (PPTX) [file pcbi.1006601.s002.pptx]

## Slide 1
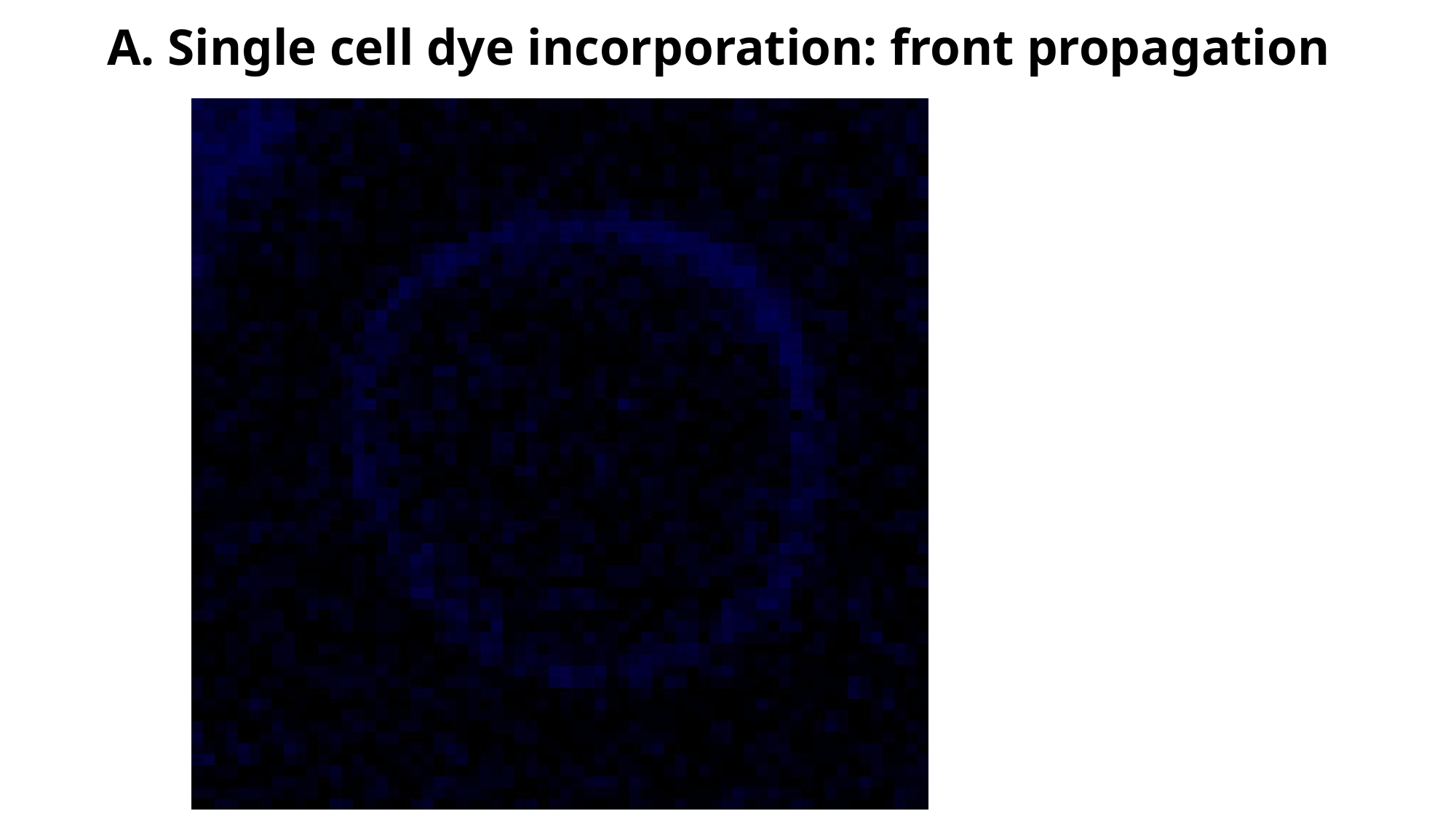

# A. Single cell dye incorporation: front propagation
20X
3 mins/frame

## Slide 2
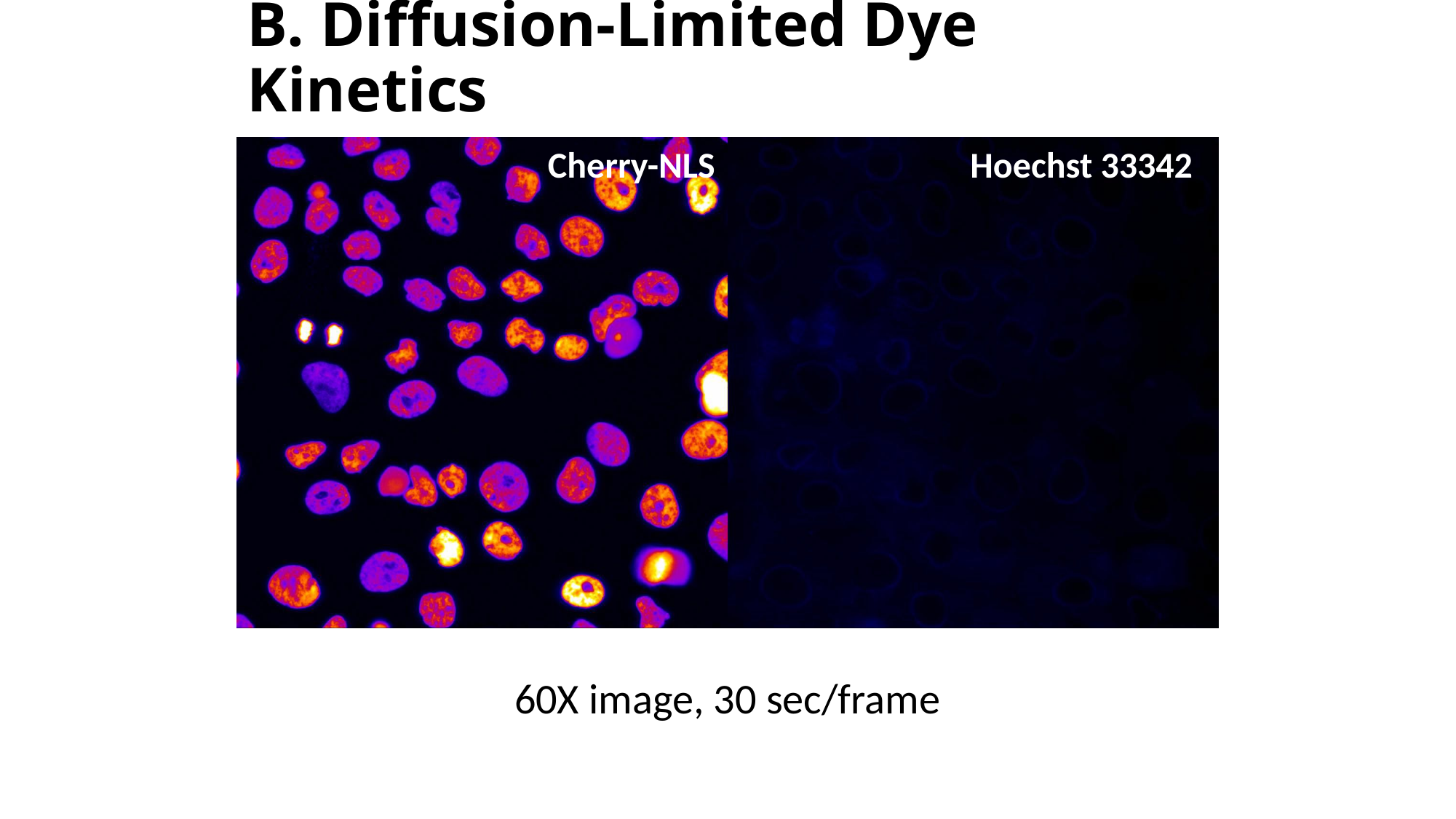

# B. Diffusion-Limited Dye Kinetics
Cherry-NLS
Hoechst 33342
60X image, 30 sec/frame

## Slide 3
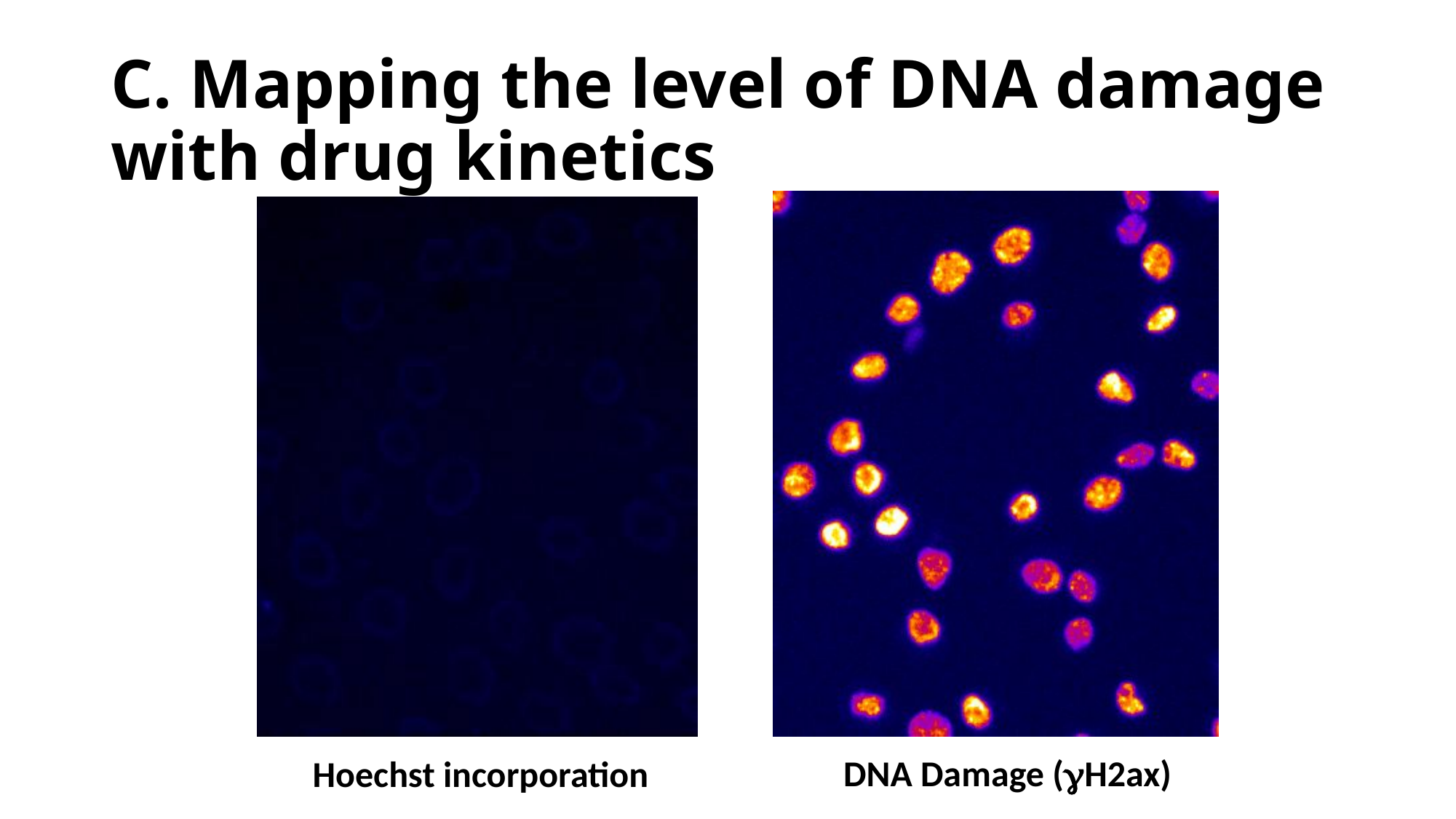

# C. Mapping the level of DNA damage with drug kinetics
DNA Damage (gH2ax)
Hoechst incorporation
